# Spatio-temporal dynamics of bacterial communities in the shoreline of Laurentian great Lake Erie and Lake St. Clair’s large freshwater ecosystems

**DOI:** 10.1186/s12866-021-02306-y

**Published:** 2021-09-21

**Authors:** Abdolrazagh Hashemi Shahraki, Subba Rao Chaganti, Daniel Heath

**Affiliations:** 1 Great Lakes Institute for Environmental Research, University of Windsor, Windsor, Ontario Canada; 2 Present Address: Cooperative Institute for Great Lakes Research, School of Environmental and Sustainability, University of Michigan, Ann Arbor, MI USA; 3 Department of Integrative Biology, University of Windsor, Windsor, Ontario Canada

**Keywords:** Bacterial community, Freshwater, Temporal variation, Spatial variation, 16S rRNA metabarcoding, Lake Erie, Lake St. Clair

## Abstract

**Background:**

Long-term trends in freshwater bacterial community composition (BCC) and dynamics are not yet well characterized, particularly in large lake ecosystems. We addressed this gap by temporally (15 months) and spatially (6 sampling locations) characterizing BCC variation in lakes Erie and St. Clair; two connected ecosystems in the Laurentian Great Lakes.

**Results:**

We found a spatial variation of the BCC between the two lakes and among the sampling locations (significant changes in the relative abundance of 16% of the identified OTUs at the sampling location level). We observed five distinct temporal clusters (UPGMA broad-scale temporal variation) corresponding to seasonal variation over the 15 months of sampling. Temporal variation among months was high, with significant variation in the relative abundance of 69% of the OTUs. We identified significant differences in taxonomic composition between summer months of 2016 and 2017, with a corresponding significant reduction in the diversity of BCC in summer 2017.

**Conclusions:**

As bacteria play a key role in biogeochemical cycling, and hence in healthy ecosystem function our study defines the scope for temporal and spatial variation in large lake ecosystems. Our data also show that freshwater BCC could serve as an effective proxy and monitoring tool to access large lake health.

**Supplementary Information:**

The online version contains supplementary material available at 10.1186/s12866-021-02306-y.

## Background

The Laurentian Great Lakes (LGLs) in North America differ markedly in their hydraulic residence time, annual lake surface temperatures, ice cover and extent, and primary production levels [1]. The LGLs are warming rapidly, and thus are highly susceptible and responsive to any added anthropogenic induced stressors [2]. Lake Erie, the smallest and shallowest of the LGLs, has undergone dramatic swings in water quality over the past century due to nutrient loading (primarily phosphates) from agricultural and urban sources [3]. Phosphate removal programs ultimately resulted in significant improvement in the state of Lake Erie [4]. However, key ecosystem services such as drinking water (for ~ 11 million people), important aquatic species habitat, water for the industrial sector and tourism/recreational activities (boating, shipping and fisheries; >$50 billion annually) are currently threatened by frequent cyanobacterial harmful algal blooms (cHABs) and hypoxia [5]. Lake St. Clair is also heavily impacted by densely populated urban areas, and because of its location upstream [6]. Lake St. Clair is very shallow and highly affected by recurrent eutrophication symptoms [7].

Microbes play fundamental roles in transforming organic carbon and reintroducing it into the food web, thus characterizing temporal and spatial changes in bacterial community composition (BCC) can provide deeper insight into the processes and mechanisms operating in lake ecosystems and ultimately improve our basic knowledge and ability to predict BCC dynamics and function. BCC temporal variation can occur hourly [8] to seasonal [9] and interannual [10]. Cyclic abiotic factors such as light [11] and temperature [12], as well as biotic factors such as bacteriophages [13], may contribute to daily, weekly and seasonal cycles, but temporal variation goes beyond such straightforward cyclic relationships. BCC spatial variation also has reported in fine-scale (within pounds) [14] to large-scale; between the lakes [15] and in the lakes [16]. Despite these studies, the temporal (long-term continuous sampling) and spatial (between the lakes and in the lakes across multiple locations) dynamics of freshwater bacterial communities still needs more characterization in the Great Lakes.

**Fig. 1:**
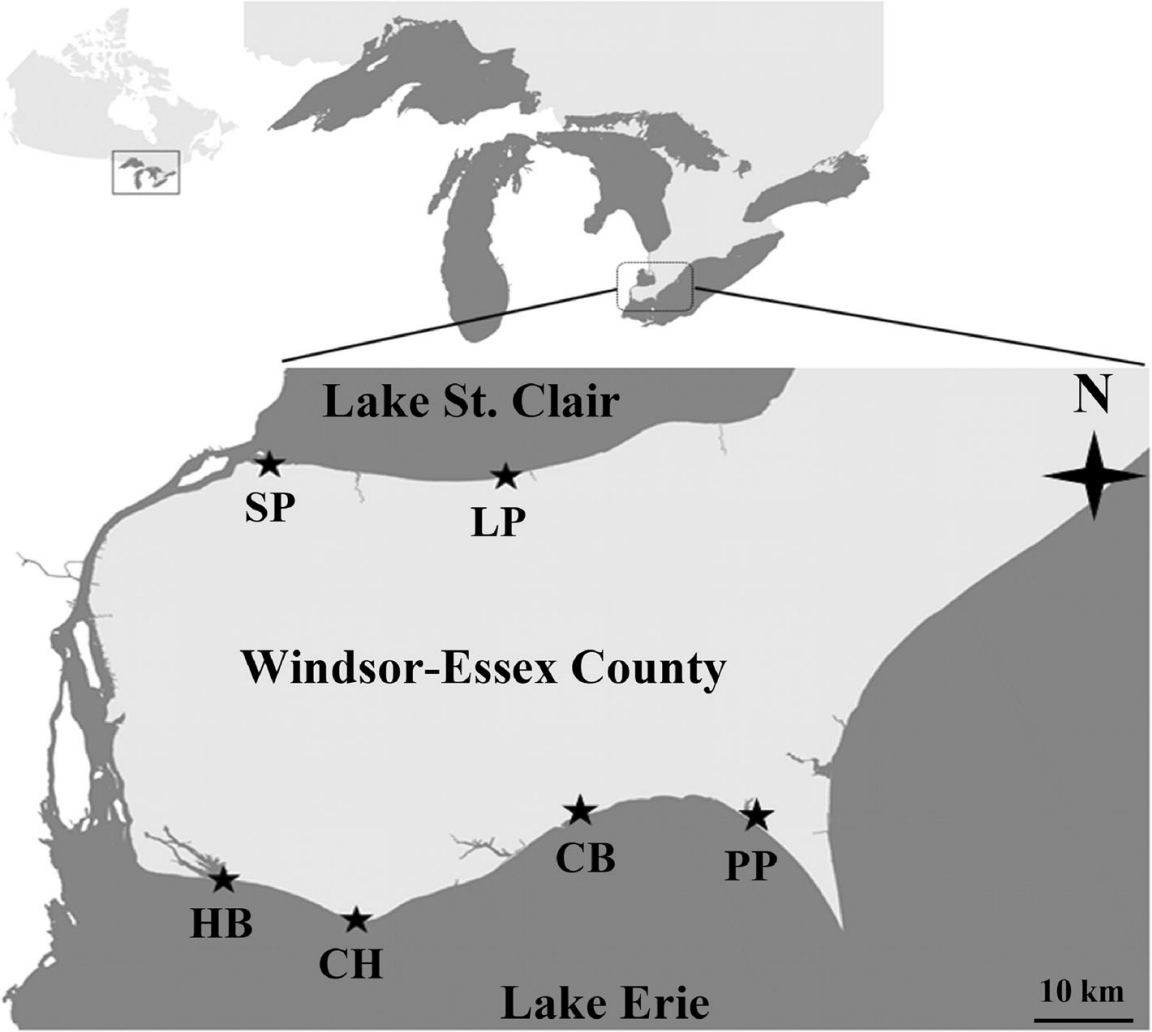
The sampling sites used for bacterial community composition in Lake Erie (Cedar Beach; CB, Colchester Harbour Beach; CH; Holiday Beach; HB and Point Pelee Beach; PP), and Lake St. Clair (Lakeview Park Beach; LP and Sand Point Beach; SP) in Windsor-Essex County. The map generated by the authors of this paper

Despite numerous studies addressing the biogeographical distribution of microbial species [8, 10, 15, 16], microbial ecologists lack a basic understanding of the characteristic scales of temporal (long-term) and spatial (between the lakes and in the lakes across multiple locations) variation in aquatic BCC; as BCC form the cornerstone of whole freshwater ecosystems. Arguably, this is a key gap in our basic understanding of aquatic bacterial diversity that hinders our ability to develop theories about how microbially mediated function and the stability of those functions are maintained across space and time. Considering previous studies [8, 10, 15, 16], we must integrate long-term temporal sampling with large scale spatial sampling to allow not only assessment of change over time and space, but also the potential for the interaction between location and date of sampling. To address this knowledge gap, we sampled bi-weekly 6 recreational beaches in Lake Erie (4 locations) and St. Clair (2 locations) at Windsor-Essex County (Windsor, Ontario, Canada) from June 2016 to August 2017 to characterize the BCC (Fig. 1). We used 16S rRNA metabarcoding via next-generation sequencing (NGS) to ensure accurate and complete BCC characterization. We hypothesize both significant temporal (bi-weekly, monthly and seasonal) and spatial (sampling location and lake) variation in freshwater BCC, given the spatial and temporal scale of this study. Specifically, we predicted stronger temporal than spatial effects, primarily due to the expected large seasonal effects, but due to environmental similarity and connectivity among the sampled sites, only subtle spatial effects. More specifically, we expected to observe highly divergent BCCs among the seasons, but with the two summer season samples more similar. We also expected that environmental parameters (water temperature, season, precipitation and day light hours) have influence on the BCC dynamic. The outcome of this study will increase our basic understanding of how freshwater BCC changes at different scales of time and space which is critical for monitoring the ecological service of BCC and aquatic ecosystem health.

## Results

### Global spatial and temporal effect

After quality control, 5.1 million Ion Torrent sequence reads remained across all 6 sample locations and 15 months. Each sample (replicate) had between 2102 and 8509 reads, with an average of 4789 reads. In total, 27,643 OTUs were detected. After removing singleton and doubleton sequence reads, as well as OTUs with ≤20 reads from the data set, 2100 OTUs were included in this study. The OTU table was rarefied to 2000 reads/sample.

We collected 2 samples per location and time and found that replicates had no significant differences in the BCC (*p* > 0.05). We also observed no significant replicate effect with 3–9 samples/location in our recently published study of aquatic bacterial community dynamics in north temperate lakes [17], thus we combined sequence read data of the two replicates for each week at each location to increase the read depth for all further statistical analyses.

**Fig. 2:**
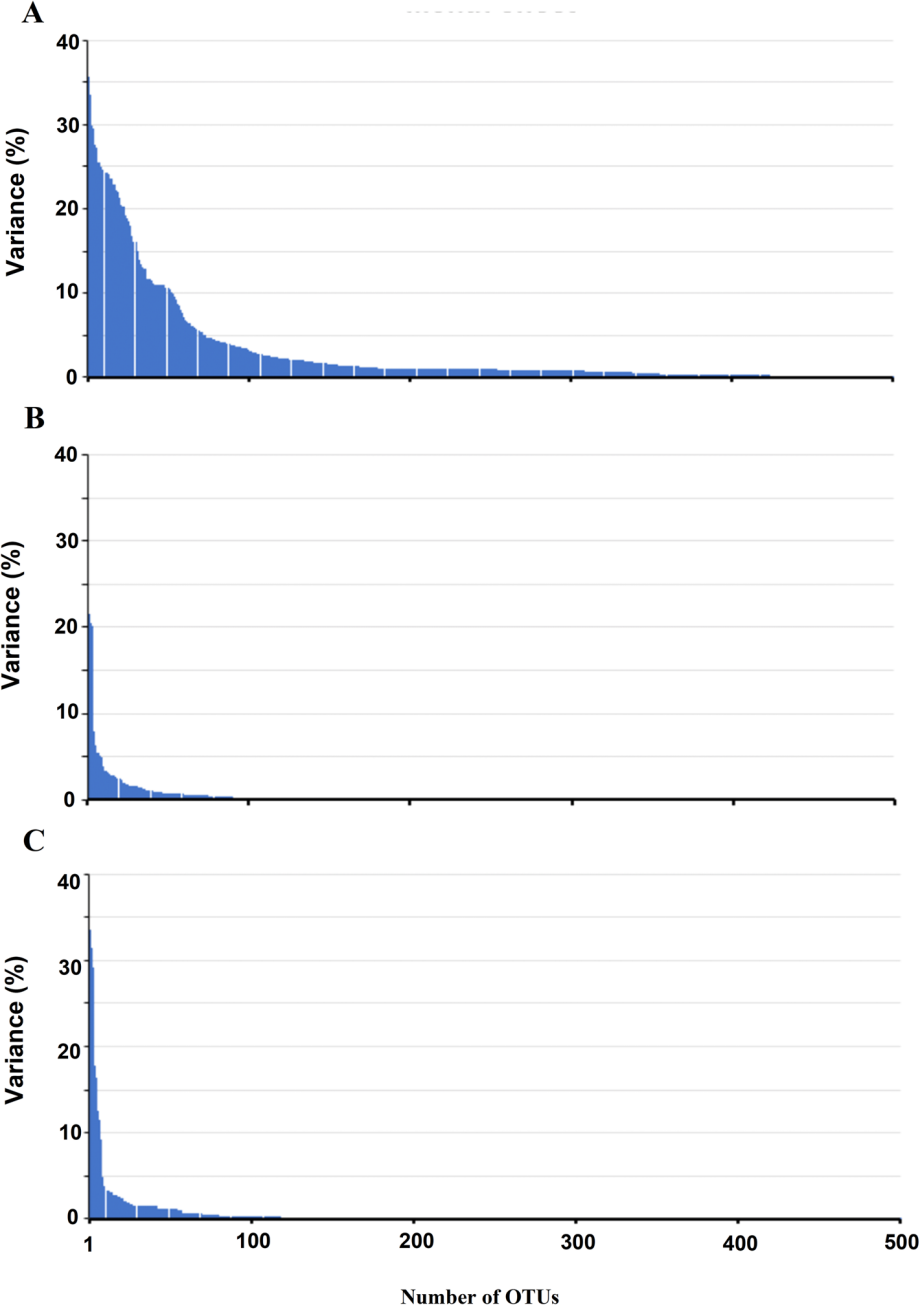
Histograms showing the effect (Kruskal–Wallis *P*-value) of location (6 sampling locations) and month (15 sampling months) and their interactions on the relative abundance of 2100 bacterial OTUs sampled at 6 sites over 15 months. Panel A; temporal effect (month), panel B; spatial effect (location) and panel C; the interaction of spatial and temporal effects (Location x Month). Uncorrected *p* values are shown on the in Y-axis and *p* = 0.05 was used as cut-off of the significant effect (dashed line in each plot)

Based on our global GLMM, we found that lake (as a broad spatial factor) had significant effects (*p* < 0.05) only on Chao1 and Shannon indexes; however, month (as a broad temporal factor) had significant effects (*p* < 0.05) on Chao1, Shannon, PCo1, PCo2 and PCo3 (Supplementary Table 1). In the lake-specific models (two models), sampling location had a significant effect (*p* < 0.05) only on Chao1 and Shannon indexes, but month had a significant impact on Chao1, Shannon, PCo1, PCo2 and PCo3 (Supplementary Table 1). The interactions of sampling location with month also had significant (p < 0.05) effects on Chao1, Shannon, PCo1, PCo2 and PCo3 in two lake-specific models (Supplementary Table 1). Our Kruskal–Wallis analyses showed that out of 2100 OTUs, the relative abundance of 336 (16%) of the OTUs were significantly affected by location (6 sampling locations), while the relative abundance of 1453 (69%) of the OTUs were significantly affected by month (15 sampling months) (Fig. 2). The interaction of location and month had a significant effect on the relative abundance of 311 (14%) OTUs (Fig. 2). Supplementary Table 2 presented top 20 OTUs which were mostly affected by month, location (6 sampling locations) and their interaction. It is important to note that significance was not corrected for multiple simultaneous comparisons; however, the goal of this analysis was to show the pattern of effects on OTU relative abundance, highlighting the dominance of temporal effects relative to spatial and interaction effects (Fig. 2).

### Spatial variation

#### Broad spatial variation

There was no significant difference in the Bray–Curtis dissimilarity matrix for the BCCs of Lake Erie and Lake St. Clair across the full sampling period (PERMANOVA; df = 1, F = 2.064, *p* = 0.07) (Supplementary Fig. 1). Similarly, we found no significant variation in the mean of the Chao1 (F = 1.2), PCo1 (F = 0.42), PCo2 (F = 0.92) and PCo3 (F = 0.94) values between two lakes by one-way ANOVA (df = 1, *p* > 0.05). Out of the 30 detected classes of bacteria across all samples, the relative abundance of only 5 of the classes was significantly higher (LDA; *p* < 0.05) in the BCCs of Lake Erie in comparison to Lake St. Clair (Supplementary Fig. 2).

#### Spatial variation among different locations

There was no significant difference in the Bray–Curtis dissimilarity matrix for the BCCs of the 6 sampling locations (PERMANOVA; df = 5, F = 1.06, *p* = 0.34) (Supplementary Fig. 1). One-way ANOVA showed a significant effect of sampling locations on the Chao1 index (F = 5.32, *p* < 0.001). Tukey post-hoc test revealed that only the mean of Chao1 index in CB was significantly higher (*p* < 0.05) than HB, PP and SP but not from CH and LP. Using one-way ANOVA, there was no significant effect of sampling locations on PCo1 (df = 5, F = 0.644, *p* = 0.66), PCo2 (df = 5, F = 0.38, *p* = 0.85) and PCo3 (df = 5, F = 0.39, *p* = 0.88). Out of the 2100 OTUs, 336 OTUs (4 highly abundant, 21 moderately abundant and 311 rare OTUs) showed significant variation among the 6 sampling locations (Fig. 2). We identified 1–5 classes of bacteria with significant divergence for some of the pairwise comparisons at the class level between the BCC of different sampling locations (Supplementary Table 3). We identified a maximum 5 classes of bacteria with significant variation in their relative abundance between CB and LP; between CB and SP; between CH and SP; between HB and SP and between LP and SP (Supplementary Table 3). Further analysis at order and family levels shown that some bacteria had common patterns among different sampling locations (Supplementary Table 3). For example, order *Aeromonadales* (phylum *Proteobacteria* and class *Gammaproteobacteria*) had significantly higher relative abundance in LP and SP in comparison to CH, HP, and PP (Supplementary Table 3). Also, order *Acidimicrobiales* (phylum *Actinobacteria* and class *Acidimicrobiia*) had significantly lower relative abundance in LP and SP in comparison to CB, CH, and PP (Supplementary Table 3).

### Temporal variation

**Fig. 3:**
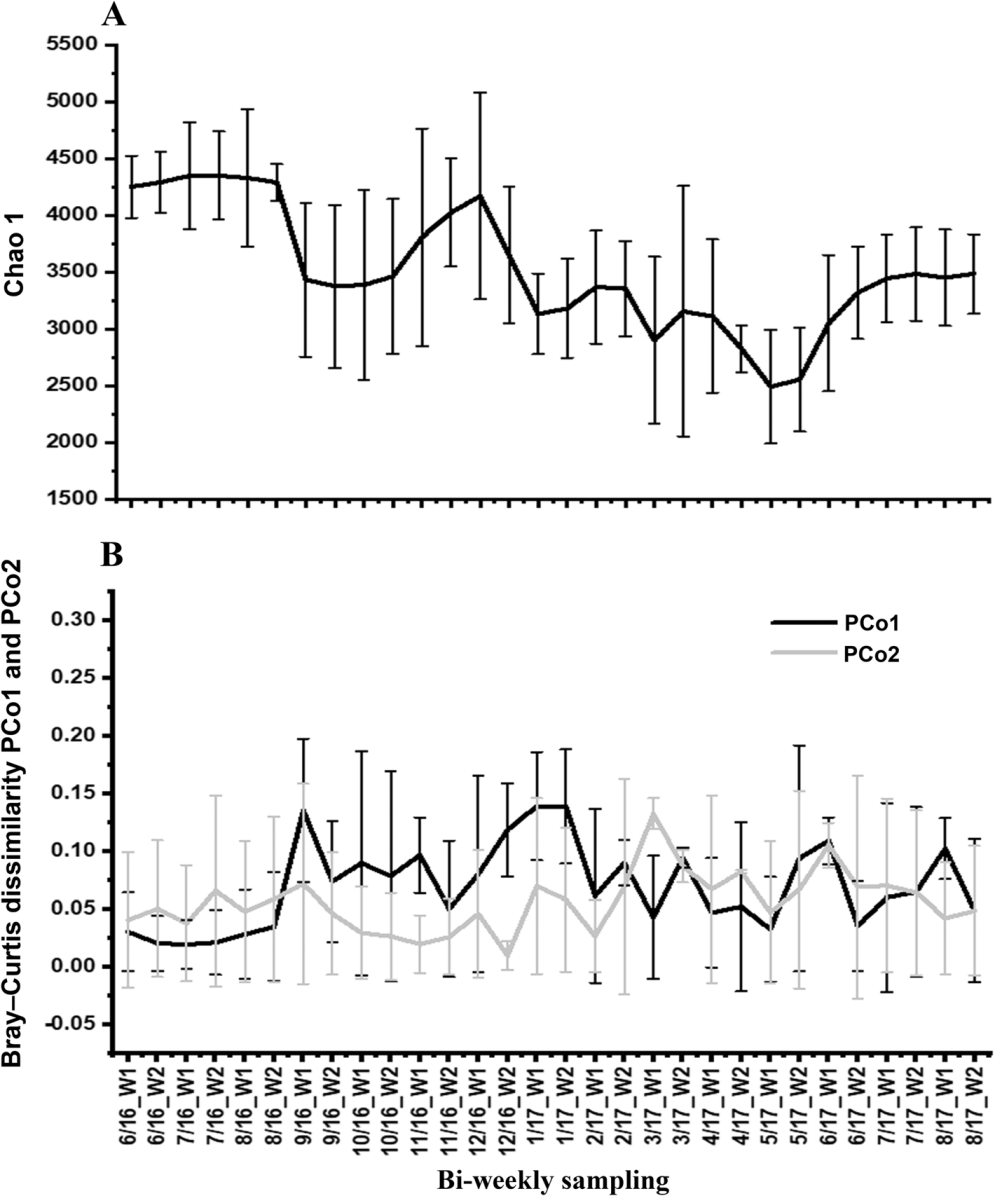
Line plots showing mean bi-weekly variation in Chao1 index (panel A) and Bray–Curtis dissimilarity PCo1 (panel B) and PCo2 (panel C) for the six different sampling locations (CB, CH, HB, LP, PP and SP) over 15 sampling months (June 2016–August 2017). Error bars show standard deviation. In X-axis; numbers indicate the month in years, W shows weeks 1 and 2; 16 and 17 indicate 2016 and 2017 years respectively

PCo1 (16.1%) and PCo2 (8.1%) (Fig. 3) and PCo3 (6.5%) (not shown), which represented the most variation in the BCC PCoA, varied substantially over the 15 months sampling period, while PCo4 (3.5%), PCo5 (3.4), PCo6 (2.2%) and PCo7 (1.5%) represented only minor levels of variation in the bi-weekly BCCs. Inspection of the bi-weekly plots of alpha diversity also shows substantial temporal variation throughout the 15-month sampling period (Fig. 3 and Supplementary Fig. 3).

### Broad temporal variation

#### BCCs variation

**Fig. 4:**
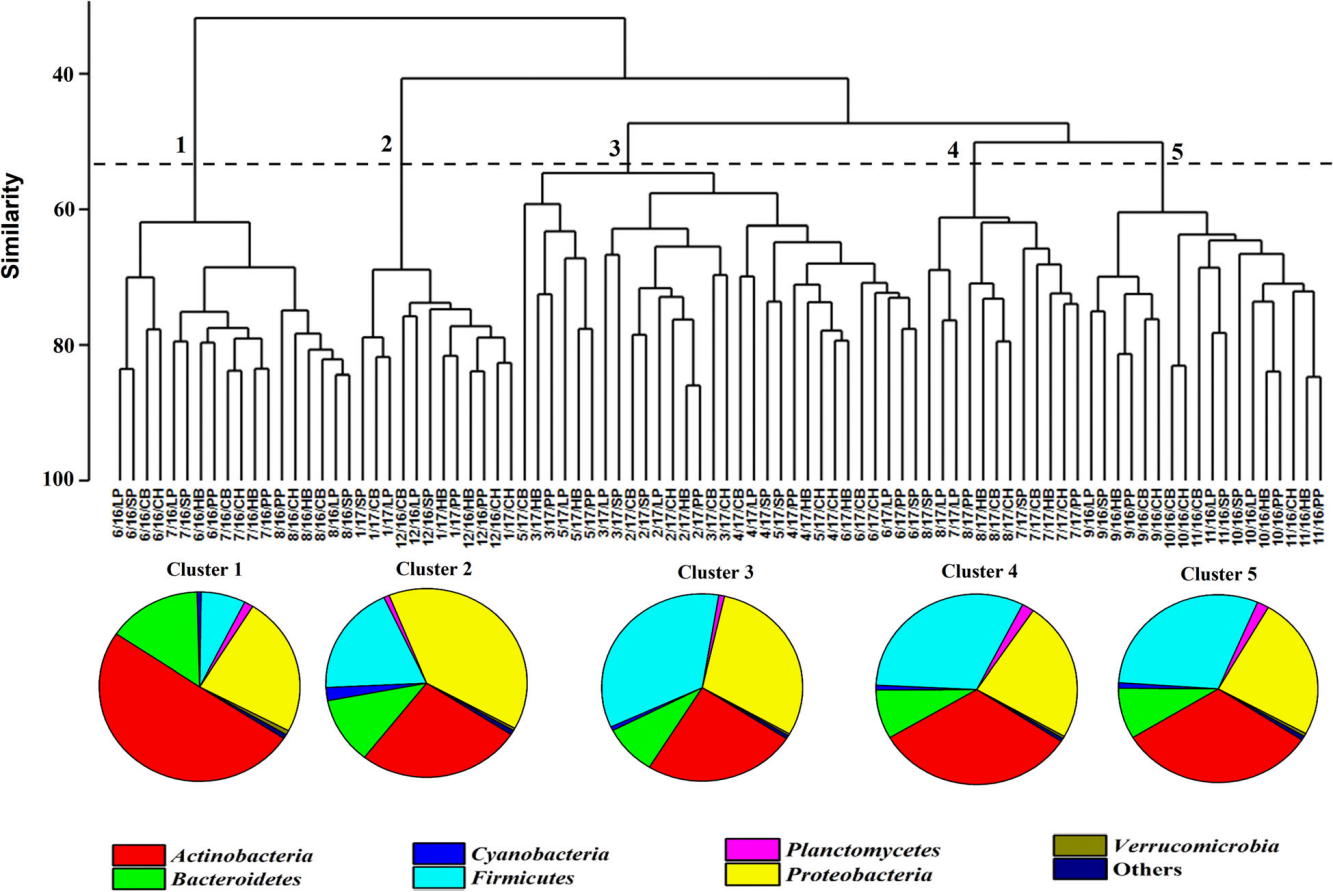
UPGMA tree showing cluster results based on Bray-Curtis similarity of the 15 monthly BCCs sampling across six sample sites (X-axis: 15 sampling months collected from 6 different location; CB, CH, HB, LP, PP and SP over 2016 and 2017. 2016 and 2017 showed as 16 and 17 respectively in the figure). The BCCs grouped into five broad temporal community clusters, all clusters were above 50–60% Bray–Curtis similarity (Y-axis) and show considerable taxonomic divergence (pie charts showing phyla level composition). For this analysis, the Bray–Curtis similarity matrix was generated using the 300 most abundant OTUs

As we were only interested in long term temporal variation, we combined bi-weekly data from each location within each month. We thus had a total of 90 samples (15 months × 6 locations) for our cluster analysis. UPGMA clustering showed five major clusters diverging at between 50 and 60% similarity based on the Bray–Curtis similarity index (Fig. 4). The BCCs of summer 2016 (June, July and August) were grouped in cluster 1. The BCCs of December (2016) and January (2017) were grouped as cluster 2. The BCCs of 5 months including February, March, April, May and June 2017 clustered together as cluster 3. The BCCs of July and August (summer 2017) were grouped as cluster 4 and the BCCs of fall 2016 (September, October and November) grouped into cluster 5 (Fig. 4). There was significant variation among the BCCs of 5 clusters (PERMANOVA test; df = 4, F = 9.57, *p* = 0.0001) and their pairwise comparisons. The overall average dissimilarity of the BCCs of 5 broad clusters was 51% using SIMPER analysis (Supplementary Table 4).

#### Diversity variation among clusters

**Fig. 5:**
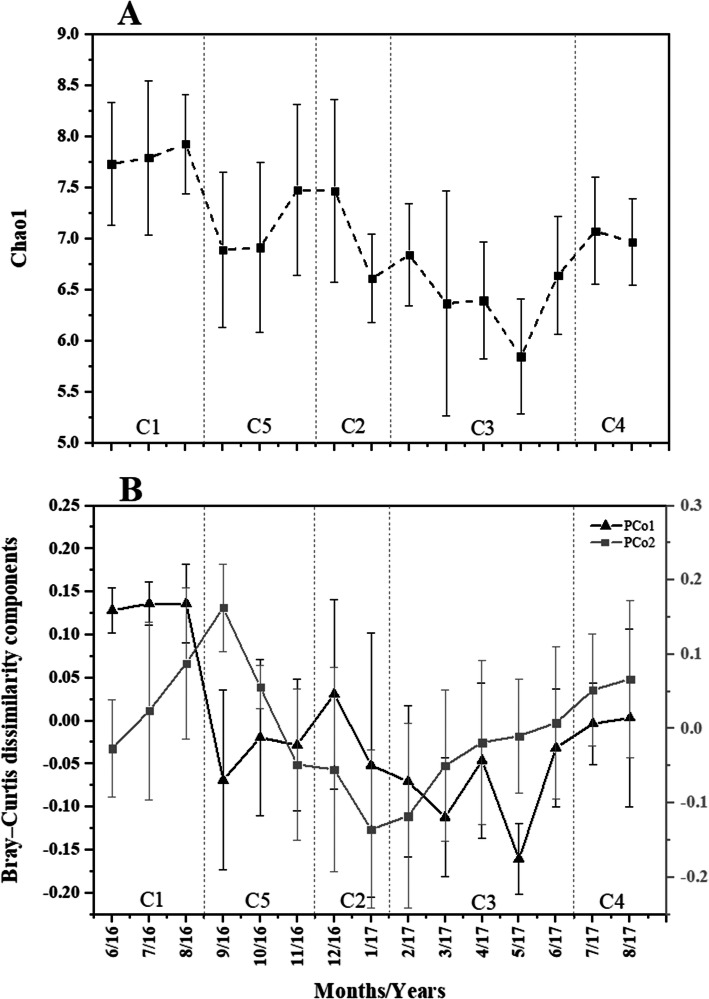
Line plots of monthly changes of Chao1 (top panel) and Bray–Curtis dissimilarity components (bottom panel) of 6 different locations over 15 months of sampling. C1–5: Cluster 1–5 is based on Fig. 4. PCo1 and 2 explained 22.9 and 14% variances of the BCC variation over 15 months respectively. X-axis: numbers indicate sampling months, 16 and 17 show sampling years 2016 and 2017 respectively

The clusters differed significantly (df = 4, *p* < 0.0001) in Chao1 (F = 27.42), PCo1 (F = 28.2,), PCo2 (F = 27.9) and PCo3 (F = 20.2) using one-way ANOVA. Figure 5 presented variation of Chao1, PCo1 and PCo2 over 15 months of sampling. Tukey post-hoc analysis showing that Chao1 and PCo1 of cluster 1 were significantly (*p* < 0.05) higher than all 4 other clusters. PCo2 of cluster 2 was significantly (p < 0.05) lower than all 4 other clusters. More interestingly, the mean of Chao1 for cluster 3 was significantly lower than all other clusters and the mean of PCo1 also was the lowest among all clusters but significantly lower than clusters 1 and 4 (Supplementary Fig. 4 and Supplementary Table 5).

#### OTUs and taxonomic variation

**Fig. 6:**
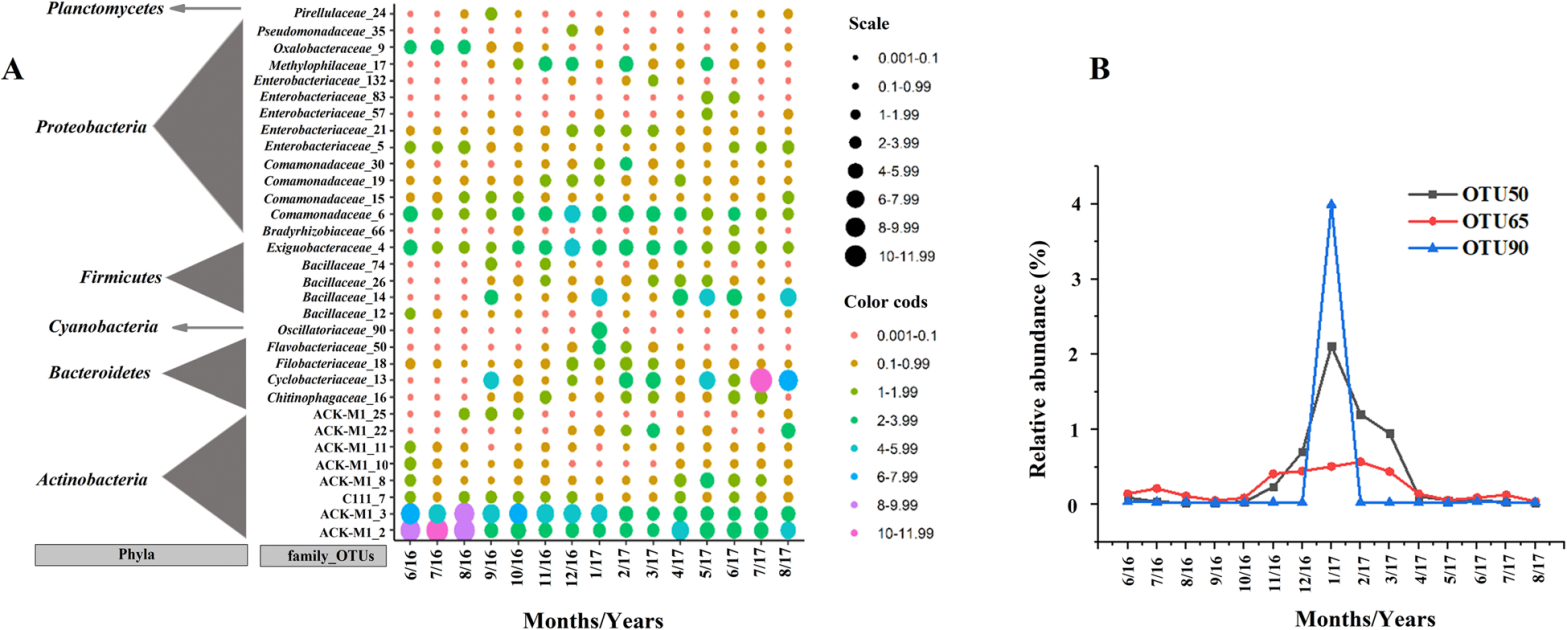
The relative abundance of highly abundant OTUs (panel A) and *Flavobacteriaceae* (OTU50) and *Oscillatoriaceae* (OTUs 65 and 90) (panel B) over 15 months of sampling

At the OTU level, clusters 1, 2, 3, 4 and 5 had 7, 12, 11, 9 and 11 highly abundant (relative abundance > 1%) OTUs, respectively. Only four OTUs (2, 3, 4 and 6) were common among all 5 clusters. Taxa information of the abundant OTUs in the clusters were presented in Fig. 6. Only OTUs 2 and 3 (phylum *Actinobacteria* and family ACK-M1), OTU4 (phylum *Firmicutes* and family *Exiguobacteraceae*) and OTU6 (phylum *Proteobacteria* and family *Comamonadaceae*) were abundant across all clusters. The relative abundance of many OTUs significantly varied among the clusters, as expected given that the clusters were defined based on variation in BCC. For example, the relative abundance of OTU2 (family ACK-M1) was significantly higher in the BCC of cluster 1 (9.5%) than all other clusters (2.5–3.8%). Out of 2100 OTUs; 75, 90, 79, 61 and 79 moderately abundant OTUs were detected in the BCCs of clusters 1, 2, 3, 4 and 5, respectively.

While the BCCs of the clusters (1–5) were dominated by four phyla, the relative proportions of these phyla varied substantially (Fig. 4). *Actinobacteria* was the most common taxon (~ 50%) versus *Firmicutes* (7.16%) as the less common taxon in the BCC of cluster 1. In the BCC of clusters 4 and 5, *Actinobacteria* (~ 32% in both clusters 4 and 5) was dominant but *Firmicutes* (~ 32% in cluster 4 and ~ 30% in cluster 5) became the second most common taxon. In the BCC of cluster 2, *Proteobacteria* (~ 39%) was the most common taxon and *Firmicutes* (~ 19%) became the third most common phylum after *Actinobacteria* (~ 26%). More interestingly, *Cyanobacteria* levels were elevated in the BCC of cluster 2 (2.25%) compared to the other clusters (0.75 ± 0.35%). The BCC of cluster 3 was enriched for *Firmicutes* (~ 35%) as the dominant phylum and by *Proteobacteria* (~ 30%) as the second most common phylum. We observed substantial variation in the relative abundance of 30 classes of bacteria. For example, the relative abundance of *Bacilli* was significantly lower in the BCC of cluster 1 compared to all other clusters, while some classes such as *Actinobacteria* and *Thermoleophilia* (belonging to *Actinobacteria*) and *Cytophagia* and *Saprospirae* (belonging to *Bacteroidetes*) conversely had significantly higher relative abundance in the BCC of cluster 1 relative to all the other clusters (Supplementary Table 6).

### Monthly temporal variation

#### BCCs variation

There was a statistically significant effect of sampling month (df = 14, F = 6.1 and *p* = 0.0001) on the Bray–Curtis dissimilarity matrix of the BCCs based on PERMANOVA. The pairwise comparison of the BCCs of the months showed significant differences for most comparisons (Supplementary Table 7). For example, there was no significant variation between the BCC of April and May (2017) in comparison to the BCC of March (2017) and the BCC of May and June (2017) in comparison to the BCC of April (2017).

#### Diversity variation

We observed significant variation (df = 14, *p* < 0.001) in Chao1 (F = 8.97), PCo1 (F = 5.7), PCo2 (F = 4.5) and PCo3 (F = 3.9) indexes among the 15 months of sampling using one-way ANOVA. Tukey post-hoc tests showed that 30% of the comparisons among sampling months were statistically significant (*p* < 0.05) for Chao1 among 15 months of sampling, while 10% of the Tukey post-hoc test comparisons of PCo1 and only 3% of the Tukey post-hoc test comparisons of PCo2 showed significant divergence (p < 0.05) (Fig. 5, Supplementary Table 8).

#### OTUs and taxa variation

Out of 2100 OTUs, the relative abundance of 1453 (69%) OTUs changed significantly (Fig. 2) across the months. The BCCs of July (2016) had the lowest number (6 OTUs) of highly abundant OTUs while the BCCs of February (2017) and June (2017) had the highest number of highly abundant OTUs (12 OTUs) among the 15 months of sampling. OTUs 2, 3 (family ACK-M1), 4 (family *Exiguobacteraceae*) and 6 (family *Comamonadaceae*) were highly abundant across all 15 months of sampling, but the relative abundance of others with high relative abundance dropped to ≤1% in some months (Fig. 6). Some highly abundant OTUs showed highly variable patterns over 15 months of sampling. For example, the relative abundance of OTU2 was 8.2, 10.8 and 9.5% in June, July and August (2016) respectively but dropped to between 2.0 and 5.7% across all other months. In contrast, the relative abundance of OTU13 (phylum *Bacteroidetes* and family *Cyclobacteriaceae*) was 0.02–4.4% in the BCCs of June 2016 – June 2017 but increased up to 10.7 and 7.2% in the BCCs of July and August (2017) respectively (Fig. 6A). Many of even the highly abundant taxa exhibited unpredictable variation across the study period, highlighting the chaotic nature of BCCs. Interestingly, the relative abundance of family *Flavobacteriaceae* belonging to *Bacteroidetes* (OTU50) and *Oscillatoriaceae* belonging to *Cyanobacteria* (OTUs 65 and 90) were elevated in the January BCCs relative to all other months (Fig. 6B).

At the phyla level, the BCCs of June (47.56%), July (49.83%) and August (53.37%) in the summer of 2016, were enriched by *Actinobacteria* compared to June (27.47%), July (30%) and August (34.78%) in summer 2017 and all other months. In contrast, taxa belong to *Firmicutes* was not common in the BCCs of June (6.93%), July (7.24%) and August (7.31%) in summer 2016 while they were consistently common in the composition of the BCCs in other months (30.43 ± 6.88%). The relative abundance of taxa belonging to class *Proteobacteria* increased in January (34.72%) and February (43.34%) compared to other months (26.21 ± 3.84%).

At the class level, significant variation (*p* < 0.05) was detected among months by pairwise LDA among the 30 identified taxonomic classes. Only 2 bacterial classes showed significant differences between the BCCs of June and July (2016), meanwhile, 24 bacterial classes showed significant variation between the BCCs of July 2016 and June 2017. Classes *Actinobacteria*, *Acidimicrobiia*, *Thermoleophilia* (phylum *Actinobacteria*) and *Saprospirae* and *Cytophagia* (phylum *Bacteroidetes*) showed high relative abundance in the June and July and August (2016) BCCs compared to other months (Supplementary Fig. 5). Conversely, class *Bacilli* had low relative abundance in the June and July and August (2016) BCCs compared to all other sampling months (Supplementary Fig. 5). *Chloroplast* became a highly abundant taxa in the BCCs of cold months (November and December; 2016 and January; 2017). We also observed a noticeable increase in the relative abundance of classes *Beta*, *Delta* and *Gammaproteobacteria* in the BCC of January (2017) and a shift in the relative abundance of class *Planctomycetia* from highly to moderately abundant in the cold months (November–May) (Supplementary Fig. 5).

Surveys of heterotrophic bacteria revealed significant changes in the composition of heterotrophic bacteria between the two summers (2016 and 2017). The change was characterized by significant reductions in the relative abundance of families such as C111 and ACK-M1 (*Actinobacteria*) in summer 2017 relative to summer 2016, coupled with increased abundance of *Cyclobacteriaceae* (*Bacteroidetes*), *Enterobacteriaceae* (*Gammaproteobacteria*) and *Bacillaceae* (*Bacilli*) in summer 2017 (Supplementary Fig. 6). Surveys of phototrophic bacteria revealed noticeably higher abundance of *Oscillatoriaceae* (*Cyanobacteria*) in colder and lower sunlight months which coincided with an increase in the abundance of *Flavobacteriaceae* family (a heterotrophic bacteria) in the colder months; November–March (Supplementary Fig. 6).

#### Influences of environmental parameters

The mean water temperature varied considerably, as high as 24.3 ± 0.4 °C and 22 ± 0.2 °C for summer 2016 and 2017 respectively, with the highest and lowest water temperatures in August 2016 (28 ± 0.72 °C) and January 2017 (0.05 ± 0.070 °C) (Supplementary Fig. 7). Environmental parameters (daylight, precipitation, water temperature and season) correlated with the BCCs of 6 sampling locations over 15 sampling months (Spearman Rho = 0.30, *p* < 0.05). Daylight (4%), precipitation (4%) and water temperature (13%) together explained 21% of the total variation of the biological data (Supplementary Fig. 8).

## Discussion

Our results showed spatial variation (between lakes and among sampling locations). It has been reported that variation of environmental parameters such as salinity, redox conditions and dissolved organic matters (DOM), etc. [18], as well as habitat variation [14], among sampling locations, are strong drivers of the spatial variation of the microbial communities. We did not measure abiotic parameters such as nutrient levels at our sampling locations; however, due to the connectivity of the two lakes by the Detroit River [19], the short distances among the sampling locations and the eutrophication of Lake Erie [5] and St. Clair [20], our sampling locations might have similar habitat features which consequently resulted in the little spatial variation of the BCCs in our study. The chao 1 index of CB was higher than other sampling sites, potentially due to presence of more greenhouse agriculture area and consequently higher bioavailability of nutrients; however, more studies are needed to address the influence of greenhouse agriculture area on BC diversity. Comparisons of upper Great Lakes (lakes Superior and Huron) BCC data with Lake Erie returned only a few OTUs (383 out of ~ 13,000 OTUs) with significant differences in their abundance [16], consistent with our observation of only relatively minor spatial variation effects on BCC sampled at the same time. A report of only minor spatial variation in the metabolic profiles in 8 carbon substrates out of 31 in the sediment microbial communities of Lake St. Clair [21] highlighted the lack of substantial spatial variation among sites even at the functional level. Three distinctly different bacterial assemblages were reported in the upper, middle and lower Yenisei River (1800 km) [22] – that study also reported matching nutrient spatial variation. The reported presence of distinct BCCs across large spatial scales versus our weak spatial effects across short distances may reflect nutrients gradients that may occur across long distance and drives microbial habitat variation.

We observed strong temporal variation over our sampling effort that included more than a year (15 months), which captured vast temporal (seasonal) variation. Clustering of the BCCs for the 15 sampling months resulted in five highly divergent BCC clades, which closely matched to seasonal patterns. Many studies have reported high prokaryotic microbial diversity in summer relative to winter [23], which we also observed. More interestingly, we found significant variation between the BCC of summer 2016 and 2017, with a significant decrease in the diversity indexes of the BCC of summer 2017. As the water temperature of two summers was not significantly different, the variation of the two summer BCCs is likely related to other abiotic (such nutrients bioavailability) and biotic factors that differed between the two summers [24, 25]. Perhaps not surprisingly, we found significant correlations between selected environmental factors (daylight hours, precipitation, water temperature and season/cluster) and the BCC; however, all these environmental factors only explained 30% of the total variation in BCC across the 15 months. Our environmental factors are indeed confounded with our temporal variation (so, for example daylight, precipitation and temperature all co-vary with season), indicating that the covariance of the environmental factors with the temporal variation (season/cluster) may reflect the mechanisms for the temporal effects. We did not measure the nutrients, oxygen content or other environmental parameters in this study which could be consider one of the limitations of this study. It will be interesting to measure those environmental parameters to better understand the reasons behind the BCC variation with the time and locations.

This study was not designed to test for annual effects; however, significant differences in the BCC in the two sampled summers (2016 and 2017) highlights the potential for unpredicted annual temporal variation along with a seasonal and monthly temporal variation of freshwater BCCs. Few studies have characterized monthly temporal variation in freshwater BCC. However, one study reported monthly monitoring of BCC of Lake Taihu over 3 years (2009–2011) at four different sites, and showed significant monthly (and consequently seasonal) variation of diversity indexes of the BCC [26]. In line with our observation of strong seasonal variation in BCC, drastic seasonal transitions of microbial abundance and diversity have been reported in lakes [27]. Reported high levels of variability in BCC of bog lakes over 5 years (with unique communities in each year of sampling) [28] was also consistent with our limited results regarding annual (summer) BCC variation in large freshwater lake ecosystems.

Few microbial taxa showed higher abundance in Lake Erie relative to Lake St. Clair, despite Lake Erie is a substantially larger and deeper lake. The few that were more abundant in Lake Erie mostly belong to *Actinobacteria*. *Actinobacteria* are often the numerically dominant phylum in lakes [25], but their abundance decreases with oxygen limitation [29] and overloading of the nutrients [30]. Low abundance of this phylum in Lake St. Clair is likely due to the low level of oxygen or higher loading of nutrients in the smaller, shallower Lake St. Clair. We found little evidence for taxonomic variation (at the class level) among sampling locations in Lake Erie perhaps reflecting relatively uniform microbial habitat characteristics among the three sampling locations (all public beaches). Interestingly, the abundance of *Bacteroidetes and Verrucomicrobia (two taxa* associated with high-nutrient environments) [25] and *Planctomycetes (a key taxon in* anaerobic ammonium oxidation) [31] were significantly different between the two sampling locations in Lake St. Clair potentially due to overloading of nutrients from an adjacent urban tributary near one of the sampling sites (LP).

*Actinobacteria* has been reported as an important components of the microbial community in Lake Erie, particularly in the summer [32] which matched our finding for 2016 but not in 2017. The summer 2017 BCC showed a significant reduction of *Actinobacteria* compared to summer 2016, potentially due to changes in abiotic variables such as reduced oxygen levels [29] or nutrient overloading [30] in summer 2017. Cyclic high abundance of *Actinobacteria* within the bacterioplankton (89%) has been reported recently by late spring in years 2013 and 2014 in parallel to high abundance of zooplankton grazing at Astatic soda pans [33]. The abundance of *Actinobacteria* did not show cyclic pattern among 2 years (summer 2016 and 2017) in our study but decreased from summer 2016 to summer 2017 continuously which potentially could not relate to the zooplankton grazing. OTUs belonging to *Proteobacteria* were in the top three most abundant phyla in the BCCs across different months and seasons. For example, *Proteobacteria* was the most abundant phylum in January and June (2017); the second most common in summer 2016 (June, July and August) and third most common in July (2017). *Proteobacteria* are reported as very abundant in many different freshwater lake habitats, but their relative abundance varies among lakes, within lakes and over time [25]. Curiously, one ubiquitous group of metabolically versatile bacterial was observed at high abundance in the coldest months of our study; order *Pseudomonadales* (such as families *Moraxellaceae* and *Pseudomonadaceae*) in January and February and *Enterobacteriaceae* (such as *Gammaproteobacteria*) in January, March, May, June and July (2017). Freshwater lake *Bacteroidetes* are often found in high abundance during periods following cyanobacterial blooms [25]. It has been reported that *Flavobacterium* spp. (belonging to *Bacteroidetes*) is the dominant taxa of the winter community in Lake Erie [32], and while we also found a significant elevation of *Flavobacteriaceae* over the cold months, we identified *Proteobacteria* as the dominant phylum in the winter (cluster 2). In our data set, the relative abundance of Cyanobacteria (family Oscillatoriaceae) and Bacteroidetes (family Flavobacteriaceae) exhibited correlated abundance in the BCC of November to March, likely reflecting the dependency of Bacteroidetes on the organic matter loading by Cyanobacteria [34]. Previous studies have also noted high levels of Cyanobacteria during winter months in freshwater reservoirs [35]. It has been suggested that high concentrations of overwintering vegetative Cyanobacteria cells may provide a large inoculum for blooms during warmer seasons [36], but the impact of family Oscillatoriaceae on algal bloom dynamics in Lake Erie and St. Clair is not well known.

Cytophaga is well known to be proficient in degrading biopolymers such as cellulose and chitin, part of the high molecular mass fraction of DOM [25]*. In our study, the relative abundance of Cytophaga (phylum Bacteroidetes) changed from being a rare component of the community in June, July and August (summer 2016) to high abundance in May, July and August (2017), indicating potentially elevated availability of DOM in the summer of 2017.* Although Firmicutes is generally a minor freshwater lake community taxon [25], in our study the relative abundance of OTUs belonging to Firmicutes increased over time. Indeed, this phylum became one of the most dominant phyla across all sampling points after the summer of 2016. Similar to our finding, a high abundance of Firmicutes (23%) was reported from water samples collected from freshwater public beaches (Ohio, Madison lake) [37]. In that study, Exiguobacterium and Paenisporosarcina were the most dominant Firmicutes genera [37], while in our study Bacillaceae (September 2016–August 2017) and Exiguobacteraceae (December, February and March 2016–17) were the most abundant genera in Lake Erie and St. Clair.

We noted a composition shift of the freshwater BCC from a community enriched by *Actinobacteria* (sensitive to nutrient overloading and low oxygen level) to one enriched by *Proteobacteria* (adapted to nutrient overloading) [25], *Bacteroidetes* (proficient in the degradation of complex biopolymers and DOM) [25] *and Firmicutes* (diverse metabolic capabilities and resistant to oxygen limitation) [38] over time. Although we did not measure nutrients in our study, the observed pattern of BCC change indicated likely increases in the loading of nutrients into both lakes from fall 2016 onwards. However, the mechanism(s) responsible for the observed BCC shift requires further investigation. Furthermore, we observed temporal variation in *Enterobacteriaceae* abundance; a family that includes many waterborne pathogens and fecal indicator bacteria (FIBs) [39], *Pseudomonadales*; a taxon which may act as an opportunistic pathogen in fish [40] and humans [41] and *Oscillatoriaceae (Cyanobacteria), all of* which reflect variation in potential pathogens and health risk, particularly over the summer.

## Conclusion

Our results showed that although freshwater BCC may have a cyclic seasonal or annual variation, the details of the composition of the community can change unpredictably over the temporal and spatial scales included in our study. The observed BCC variation could be linked to the functional activity of the community, making additional studies necessary to characterize the consequence of this variation on the ecological services of BCC in a large freshwater ecosystem. Our results also showed that long term monitoring of the bacterial community could serve as a sensitive proxy of freshwater ecosystems health and perhaps even function.

## Methods

### Study sites and sample collection

Freshwater samples were collected bi-weekly over 15 months from June 2016 to August 2017 from shorelines at 6 locations, including four locations from Lake Erie (Cedar Beach (CB), Colchester Harbour (CH) Beach, Holiday Beach (HB), and Point Pelee (PP) Beach) and 2 locations from Lake St. Clair (Lakeview Park (LP) Beach and Sand Point (SP) Beach) at Windsor-Essex County (Windsor, Ontario, Canada). LP and SP are in urban areas, while CB, CH, HB and PP are located in agricultural areas. Among these 4 sampling sites, CB is more impacted by greenhouse agriculture area. LP is near an urban tributary (the Belle River joins Lake St. Clair at LP beach), while SP and HB are near the inlet and outlet of the Detroit River respectively. Two water samples (each 250 mL) were collected at 0.5 m depth at each location and 2–3 m far from shore. Only for LP; the samples were collected from 3 m distance of shorline to meet 0.5 m depth of sampling. In total, we collected 60 samples (2 replicates * 2 weeks/month * 15 months = 60 samples) at each location between 8:00–11:00 AM. Water samples were transported to the laboratory on ice and were filtered using 0.2 μm polycarbonate membranes (Millipore, USA), and the filter immediately stored at − 20 °C until DNA extractions were performed. Water temperature was measured at the time of sample collection at each location. Other environmental variables, such as precipitation and daylight hours, were collected from Environment Canada (http://climate.weather.gc.ca/historical_data/search_historic_data_e.html) according to the sampling date.

### DNA extraction, PCR, library preparation and NGS

DNA was extracted following Shahraki et al. [42]. The extracted DNA was used as a template to amplify the V5-V6 region (~ 350 bp) of the 16S rRNA gene using V5F and V6R primers [43]. Then sample barcode and adaptor sequences were ligated to each PCR product by a second, ligation, PCR [43]. Second-round PCR products were pooled and purified using the QIAquick Gel Extraction Kit (QIAGEN, Toronto, ON, Canada). The library was then diluted to 60 pmol/L and sequenced on an Ion PGM™ System (Thermo Fisher Scientific, Burlington, ON, Canada) with 400-base read length chemistry.

### Bioinformatics

#### Sequence handling

We used the Quantitative Insights into Microbial Ecology (QIIME V. 1.9.1) bioinformatics pipeline to de-multiplex the sequences, quality filters and trim of the adaptor and primers [44]. Briefly, a length cut-off of 200 bp was selected for quality assurance of the sequences. We used usearch quality filter pipeline (usearch_qf script) using USEARCH integrated into QIIME V. 1.9.1 to perform filtering of noisy sequences, chimera checking, and operational taxonomic units (OTUs) picking on a set of de-multiplexed (post split_libraries.py) sequences. Sequences were initially sorted by length, and then de-replication was performed using –max_rejects = 500, followed by sorting by abundance. The sequences were clustered using 97% sequence identity to filter noisy reads. Chimera checking was performed using UCHIME and chimera-free sequences were used for OTU assignment using the Basic Local Alignment Search Tool (BLAST) against Greengenes 16S rRNA database as a reference data file [45]. The representative sequence for each OTU was selected using the most abundant method for assigning taxonomy in the Ribosomal Database Project (RDP) classifier program with a minimum 80% confidence level [46]. To minimize the impact of zero-inflation, after removing of single and double read OTUs using QIIME command (filter_otus_from_otu_table.py), we further removed the OTUs with ≤20 reads from the whole library manually. Then we used arcsine square root transformation to normalize the compositional and library size variation of the biological data (OTU table). The OTU table was rarefied to 2000 quality passed sequences for each sample to calculate alpha diversity. The original OTU table (non-rarefied) was used to calculate relative abundance. We defined an OTU as “abundant” when it had a relative abundance above 1% of the community, “moderate” when the relative abundance was between 0.1–0.99% and “rare” when the abundance was below 0.1% [47]. Original fastq files with metadata are deposited in NCBI Sequence Read Archive (ID PRJNA662419).

### Statistical analysis

#### Global spatial and temporal effects

We used nested ANOVA in R environment (version 3.1.1) [48] to determine replicate effect on the BCC. Alpha diversity indexes (Chao1 and Shannon) and the first (PCo1) and second (PCo2) principal coordinates from the principal coordinates analysis (PCoA) across all samples (including replicates) were used as a dependent variable in the nested ANOVA to test specifically for a replicate effect.

We used a generalized linear mixed model (GLMM) with Maximum Likelihood (ML) method in R environment (package lme4) [49] to test the effect of sampling location, lake (Erie and St Clair), month and the their interactions effects on Chao1, Shannon, PCo1 and PCo2 as dependent variables. Chao1 and Shannon were calculated using the rarified OTU table. The Bray–Curtis dissimilarity matrix was calculated using Primer-e software version 7.0.13 and after PCoA, we selected PCo1 (16.1%) and PCo2 (8.1%) and PCo3 (6.5%), which represented the most variation in the BCC PCoA. For each dependent variable, we run a global GLMM model by nesting sampling locations (6 locations) within the lake (2 lakes) and sampling weeks (2 weeks/month) within sampling month (15 months) to determine the effect size of month and lake on the BCCs. We used replicates as a random factor and sampling month and lake as fixed factors. To evaluate the effect of the sampling location by month interaction, we ran GLMM models on data from each lake separately. In these models, we considered month (weeks nested within 15 sampling months) and sampling locations as a fixed factor and replicates as a random factor. We also measured the impact of spatial (sampling locations) and temporal (sampling months) variation on the relative abundance of each OTU (*n* = 2100) using the Kruskal–Wallis one-way analysis of variance using R environment (version 3.1.1) [48].

#### Spatial variation

To characterize BCC spatial variation (between lakes and sampling locations), we used i) permutational multivariate analysis of variance (PERMANOVA) with 9999 permutations using Primer-e software to compare the Bray–Curtis dissimilarity of the BCCs, ii) PCoA of the Bray–Curtis similarity matrix to visualize the pattern of the BCC variation using Primer-e software, iii) one-way ANOVA to compare the mean of diversity indexes and PCo1 and PCo2 of the BCCs using R environment (version 3.1.1) [48], and iv) linear discriminant analysis (LDA) using the LEfSe method [50] to compare the relative abundance of taxa (class, order and family levels) in the bacterial communities.

#### Temporal variation

We applied hierarchical agglomerative clustering on the Bray–Curtis similarity matrix of the BCCs (top 300 highly abundant OTUs) using the group average method using Primer-e software to explore the possibility of seasonal clustering in terms of BCC. Once we identified clear clusters, we tested for differences in BCC among the clusters and the 15 months of sampling following the same approach as we used for the spatial variation (see above). Moreover, we used SIMPER analysis on the Bray–Curtis dissimilarity matrix to compare the overall dissimilarity among clusters and the 15 months of sampling in the vegan package [51]. Plots and graphs were generated using R (version 3.1.1) [48].

#### Environmental effects

We applied a RELATE analysis (Spearman’s p correlation coefficient) on the Bray-Curtis similarity matrix calculated from whole data sets and the matrix of Euclidean distances calculated from normalized environmental data (daylight hours, precipitation, water temperature and season) as the environmental matrix to evaluate the relationship between the BCC and environmental factors. A distance-based linear model (distLM) was used for analyzing the relationship between the Bray-Curtis similarity matrix of the BCC and the environmental variables using Primer-e software version.

## Supplementary Information

**Additional file 1: Supplementary Fig. 1.** Multivariant principal coordinate analysis (PCoA) plot of the Bray–Curtis similarity matrix of the BCCs of Lake Erie and Lake St. Clair (Top panel) and six different locations (CB, CH, HB, LP, PP and SP) (bottom panel) across 15 months of sampling.

**Additional file 2: Supplementary Fig. 2.** Taxa with significant spatial variation in their relative abundance among two lakes (Lake Erie and St. Clair). The relative abundance of all 5 classes were significantly higher (*p* < 0.05) in Lake Eire relative to Lake St. Clair.

**Additional file 3: Supplementary Fig. 3.** Bi-weekly variation in the Shannon index for the 6 different sampling locations (CB, CH, HB, LP, PP and SP) over 15 months of sampling (June 2016–August 2017). The X-axis shows time of sampling (bi-weekly sampling).

**Additional file 4: Supplementary Fig. 4.** Line plots of monthly changes of A) Shannon and B) Bray–Curtis dissimilarity components; PCo3–5 of 6 different locations over 15 months of sampling. C1–5: Clusters 1–5 are based on Fig. 4.

**Additional file 5: Supplementary Fig. 5.** Bar chart showing the relative abundance of the BCCs at the class-level for combined sampling locations and bi-weekly sampling over the 15 months of sampling.

**Additional file 6: Supplementary Fig. 6.** Phylogenetic affiliations of the top heterotrophic bacterial (panel a) and phototrophic (panel b) OTU groups from the 15 months of sampling in Lake Erie and St. Clair over 2016 and 2017. Due to space constraints, only taxa that had a relative abundance of more than 1% in at least one sampling month are presented for heterotrophic bacterial (panel a).

**Additional file 7: Supplementary Fig. 7.** The pattern of environmental parameter variation (water temperature, precipitation and daylight duration) for the 6 sampling locations over 15 months of the sampling. As air and water temperature both had the same pattern spatially and temporally (no significant variation) we only plotted water temperature. For each month, 2 different weeks were sampled (weeks 1 and 2). Sampling was started in June 2016 and ended in August 2017. Error bars, showing the standard deviation of water temperature and precipitation among 6 sampling locations.

**Additional file 8: Supplementary Fig. 8.** Distance-based Redundancy Analysis (dbRDA) of freshwater microbiota. The relative position of water samples in the biplot is based on Bray Curtis similarity of arcsine square root transformed relative abundance at the OTU level. Vectors indicate the weight and direction of the environmental variables that were best predictors of the BCCs of different months as suggested by the results of the distance-based linear model (distLM). The dbRDA axes describe the percentage of the fitted or total variation explained by each axis while being constrained to account for group differences. Sample IDs indicate the sampling months.

**Additional file 9: Supplementary Table 1.** Results of GLMM analysis of bacterial community variation temporally and spatially. Dependent variables included alpha diversity indexes and Bray–Curtis dissimilarity principal coordinate analysis axes (PCo1 and PCo2). Degrees of freedom, F value and *p* values are shown (significant p values are highlighted).

**Additional file 10: Supplementary Table 2.** List of top 20 abundant OTUs affected by time, location and their interaction.

**Additional file 11: Supplementary Table 3.** Taxa which shown significant spatial variation (*p* < 0.05) in the six sampling locations in Lakes Erie and St Clair.

**Additional file 12: Supplementary Table 4.** SIMPER results (above the diagonal) and pairwise PERMANOVA probabilities (below the diagonal) of 5 broad clusters of the BCCs. p values were adjusted using a sequential Bonferroni correction for multiple comparisons.

**Additional file 13: Supplementary Table 5.** Pairwise comparison of diversity indexes between the BCCs of 5 clusters.

**Additional file 14: Supplementary Table 6.** Pairwise comparison of temporal variation of taxa (class level) between the BCCs of 5 clusters.

**Additional file 15: Supplementary Table 7.** Pairwise dissimilarity (%; SIMPER) (above the diagonal) and PERMANOVA significance probabilities (below the diagonal) for the BCCs across the 15 months of sampling (numbers indicate months, 16 and 17 show 2016 and 2017 respectively. *P* values were adjusted using a Bonferroni correction for multiple comparisons.

**Additional file 16: Supplementary Table 8.** Pairwise comparison of alpha diversity and Bray–Curtis dissimilarity PCo1 & 2 indexes among 15 months of sampling across 6 different locations.

## Data Availability

The datasets used and/or analysed during the current study are available from the corresponding author on reasonable request.

## Abbreviations

BCC: Bacterial community composition
LGLs: Laurentian Great Lakes
cHABs: Cyanobacterial harmful algal blooms
NGS: Next-generation sequencing
DOM: Dissolved organic matters
CB: Cedar Beach
CH: Colchester Harbour Beach
HB: Holiday Beach
PP: Point Pelee Beach
LP: Lakeview Park Beach
SP: Sand Point Beach
QIIME: Quantitative Insights into Microbial Ecology
OTUs: Operational taxonomic units
BLAST: Basic Local Alignment Search Tool
RDP: Ribosomal Database Project
PCoA: Principal coordinates analysis
GLMM: Generalized linear mixed model
ML: Maximum Likelihood
LDA: Linear discriminant analysis
distLM: Distance-based linear model

## References

[1] Sterner RW, Ostrom P, Ostrom NE, Klump JV, Steinman AD, Dreelin EA (2017). Grand challenges for research in the Laurentian Great Lakes. Limnol Oceanogr.

[2] Adrian R, O'Reilly CM, Zagarese H, Baines SB, Hessen DO, Keller W (2009). Lakes as sentinels of climate change. Limnol Oceanogr.

[3] Davis CC (1964). Evidence for the eutrophication of Lake Erie from phytoplankton records. Limnol Oceanogr.

[4] Schindler DW, Carpenter SR, Chapra SC, Hecky RE, Orihel DM (2016). Reducing phosphorus to curb lake eutrophication is a success. Environ Sci Technol.

[5] Watson SB, Miller C, Arhonditsis G, Boyer GL, Carmichael W, Charlton MN (2016). The re-eutrophication of Lake Erie: harmful algal blooms and hypoxia. Harmful Algae.

[6] Scavia D, DePinto JV, Bertani I (2016). A multi-model approach to evaluating target phosphorus loads for Lake Erie. J Great Lakes Res.

[7] Casey GD: National water-quality assessment of the Lake Erie-Lake St. Clair Basin, Michigan, Indiana, Ohio, Pennsylvania, and New York: Environmental and hydrologic setting, vol. 97: US Department of the Interior, US Geological Survey; 1998.

[8] Shahraki AH, Chaganti SR, Heath DD (2020). Diel dynamics of freshwater bacterial communities at beaches in Lake Erie and Lake St. Clair, Windsor, Ontario. Microb Ecol.

[9] Bush T, Diao M, Allen RJ, Sinnige R, Muyzer G, Huisman J (2017). Oxic-anoxic regime shifts mediated by feedbacks between biogeochemical processes and microbial community dynamics. Nat Commun.

[10] Wang H, Zhang C, Chen F, Kan J (2020). Spatial and temporal variations of bacterioplankton in the Chesapeake Bay: a re-examination with high-throughput sequencing analysis. Limnol Oceanogr.

[11] Hölker F, Wurzbacher C, Weißenborn C, Monaghan MT, Holzhauer SI, Premke K (2015). Microbial diversity and community respiration in freshwater sediments influenced by artificial light at night. Philos Trans R Soc Lond Ser B Biol Sci.

[12] Villaescusa JA, Jørgensen SE, Rochera C, Velázquez D, Quesada A, Camacho A (2016). Carbon dynamics modelization and biological community sensitivity to temperature in an oligotrophic freshwater Antarctic lake. Ecol Model.

[13] Yoshida T, Nishimura Y, Watai H, Haruki N, Morimoto D, Kaneko H (2018). Locality and diel cycling of viral production revealed by a 24 h time course cross-omics analysis in a coastal region of Japan. ISME J.

[14] Lear G, Bellamy J, Case BS, Lee JE, Buckley HL (2014). Fine-scale spatial patterns in bacterial community composition and function within freshwater ponds. ISME J.

[15] Small GE, Finlay JC, McKay R, Rozmarynowycz MJ, Brovold S, Bullerjahn GS (2016). Large differences in potential denitrification and sediment microbial communities across the Laurentian great lakes. Biogeochemistry..

[16] Rozmarynowycz MJ, Beall BF, Bullerjahn GS, Small GE, Sterner RW, Brovold SS (2019). Transitions in microbial communities along a 1600 km freshwater trophic gradient. J Great Lakes Res.

[17] Sadeghi J, Chaganti SR, Shahraki AH, Heath DD (2021). Microbial community and abiotic effects on aquatic bacterial communities in north temperate lakes. Sci Total Environ.

[18] Beck M, Reckhardt A, Amelsberg J, Bartholomä A, Brumsack H-J, Cypionka H (2017). The drivers of biogeochemistry in beach ecosystems: a cross-shore transect from the dunes to the low-water line. Mar Chem.

[19] Burniston D, Dove A, Backus S, Thompson A (2018). Nutrient concentrations and loadings in the St. Clair River–Detroit River Great Lakes Interconnecting Channel. J Great Lakes Res.

[20] Bocaniov SA, Van Cappellen P, Scavia D (2019). On the role of a large shallow lake (Lake St. Clair, USA-Canada) in modulating phosphorus loads to Lake Erie. Water Resour Res.

[21] Oest A, Alsaffar A, Fenner M, Azzopardi D, Tiquia-Arashiro SM (2018). Patterns of change in metabolic capabilities of sediment microbial communities in river and lake ecosystems. Int J Microbiol.

[22] Kolmakova OV, Gladyshev MI, Rozanov AS, Peltek SE, Trusova MY (2014). Spatial biodiversity of bacteria along the largest Arctic river determined by next-generation sequencing. FEMS Microbiol Ecol.

[23] Hao C, Wei P, Pei L, Du Z, Zhang Y, Lu Y (2017). Significant seasonal variations of microbial community in an acid mine drainage lake in Anhui Province, China. Environ Pollut.

[24] Bižić-Ionescu M, Amann R, Grossart H-P (2014). Massive regime shifts and high activity of heterotrophic bacteria in an ice-covered lake. PLoS One.

[25] Newton RJ, Jones SE, Eiler A, McMahon KD, Bertilsson S (2011). A guide to the natural history of freshwater lake bacteria. Microbiol Mol Biol Rev.

[26] Peng Y, Yue D, Xiao L, Qian X (2018). Temporal variation and co-occurrence patterns of bacterial communities in eutrophic Lake Taihu, China. Geomicrobiol J.

[27] Butler TM, Wilhelm A-C, Dwyer AC, Webb PN, Baldwin AL, Techtmann SM (2019). Microbial community dynamics during lake ice freezing. Sci Rep.

[28] Linz AM, Crary BC, Shade A, Owens S, Gilbert JA, Knight R (2017). Bacterial community composition and dynamics spanning five years in freshwater bog lakes. MSphere..

[29] Taipale S, Jones RI, Tiirola M (2009). Vertical diversity of bacteria in an oxygen-stratified humic lake, evaluated using DNA and phospholipid analyses. Aquat Microb Ecol.

[30] Haukka K, Kolmonen E, Hyder R, Hietala J, Vakkilainen K, Kairesalo T (2006). Effect of nutrient loading on bacterioplankton community composition in lake mesocosms. Microb Ecol.

[31] Wagner M, Horn M (2006). The *Planctomycetes*, *Verrucomicrobia*, *Chlamydiae* and sister phyla comprise a superphylum with biotechnological and medical relevance. Curr Opin Biotechnol.

[32] Wilhelm SW, LeCleir GR, Bullerjahn GS, McKay RM, Saxton MA, Twiss MR (2014). Seasonal changes in microbial community structure and activity imply winter production is linked to summer hypoxia in a large lake. FEMS Microbiol Ecol.

[33] Szabo A, Korponai K, Somogyi B, Vajna B, Voros L, Horvath Z (2020). Grazing pressure-induced shift in planktonic bacterial communities with the dominance of acIII-A1 actinobacterial lineage in soda pans. Sci Rep.

[34] Eiler A, Bertilsson S (2007). Flavobacteria blooms in four eutrophic lakes: linking population dynamics of freshwater bacterioplankton to resource availability. Appl Environ Microbiol.

[35] Valério E, Faria N, Paulino S, Pereira P (2008). Seasonal variation of phytoplankton and cyanobacteria composition and associated microcystins in six Portuguese freshwater reservoirs. Int J Lim.

[36] Ma J, Qin B, Paerl HW, Brookes JD, Hall NS, Shi K (2016). The persistence of cyanobacterial (*Microcystis* spp.) blooms throughout winter in Lake Taihu, China. Limnol Oceanogr.

[37] Lee CS, Kim M, Lee C, Yu Z, Lee J (2016). The microbiota of recreational freshwaters and the implications for environmental and public health. Front Microbiol.

[38] Martiny JBH, Bohannan BJ, Brown JH, Colwell RK, Fuhrman JA, Green JL (2006). Microbial biogeography: putting microorganisms on the map. Nat Rev Microbiol.

[39] Ramírez-Castillo F, Loera-Muro A, Jacques M, Garneau P, Avelar-González F, Harel J (2015). Waterborne pathogens: detection methods and challenges. Pathogens..

[40] Su L, Xu C, Cai L, Qiu N, Hou M, Wang J (2020). Susceptibility and immune responses after challenge with Flavobacterium columnare and Pseudomonas fluorescens in conventional and specific pathogen-free rare minnow (Gobiocypris rarus). Fish Shellfish Immun.

[41] Malhotra S, Hayes D, Wozniak DJ (2019). Cystic fibrosis and Pseudomonas aeruginosa: the host-microbe interface. Clin Microbiol Rev.

[42] Shahraki AH, Chaganti SR, Heath D (2019). Assessing high-throughput environmental DNA extraction methods for meta-barcode characterization of aquatic microbial communities. J Water Health.

[43] He X, Chaganti SR, Heath DD (2017). Population-specific responses to interspecific competition in the gut microbiota of two Atlantic Salmon (Salmo salar) populations. Microb Ecol.

[44] Caporaso JG, Kuczynski J, Stombaugh J, Bittinger K, Bushman FD, Costello EK (2010). QIIME allows analysis of high-throughput community sequencing data. Nat Methods.

[45] Edgar RC (2010). Search and clustering orders of magnitude faster than BLAST. Bioinformatics..

[46] Wang Q, Garrity GM, Tiedje JM, Cole JR (2007). Naive Bayesian classifier for rapid assignment of rRNA sequences into the new bacterial taxonomy. Appl Environ Microbiol.

[47] Logares R, Audic S, Bass D, Bittner L, Boutte C, Christen R (2014). Patterns of rare and abundant marine microbial eukaryotes. Curr Biol.

[48] Team RC (2013). R: a language and environment for statistical computing.

[49] Bates D, Mächler M, Bolker B, Walker S: Fitting linear mixed-effects models using lme4. arXiv preprint arXiv:14065823. 2014.

[50] Segata N, Izard J, Waldron L, Gevers D, Miropolsky L, Garrett WS (2011). Metagenomic biomarker discovery and explanation. Genome Biol.

[51] Dixon P (2003). VEGAN, a package of R functions for community ecology. J Veg Sci.

