# Supplementary material for: Spatio-temporal dynamics of bacterial communities in the shoreline of Laurentian great Lake Erie and Lake St. Clair’s large freshwater ecosystems
Source: BMC Microbiol. 2021 Sep 21;21:253. doi: 10.1186/s12866-021-02306-y (PMC8454060; doi:10.1186/s12866-021-02306-y)
Supplement: Supplementary file 1 — Additional file 1: Supplementary Fig. 1. Multivariant principal coordinate analysis (PCoA) plot of the Bray–Curtis similarity matrix of the BCCs of Lake Erie and Lake St. Clair (Top panel) and six different locations (CB, CH, HB, LP, PP and SP) (bottom panel) across 15 months of sampling. [file 12866_2021_2306_MOESM1_ESM.docx]

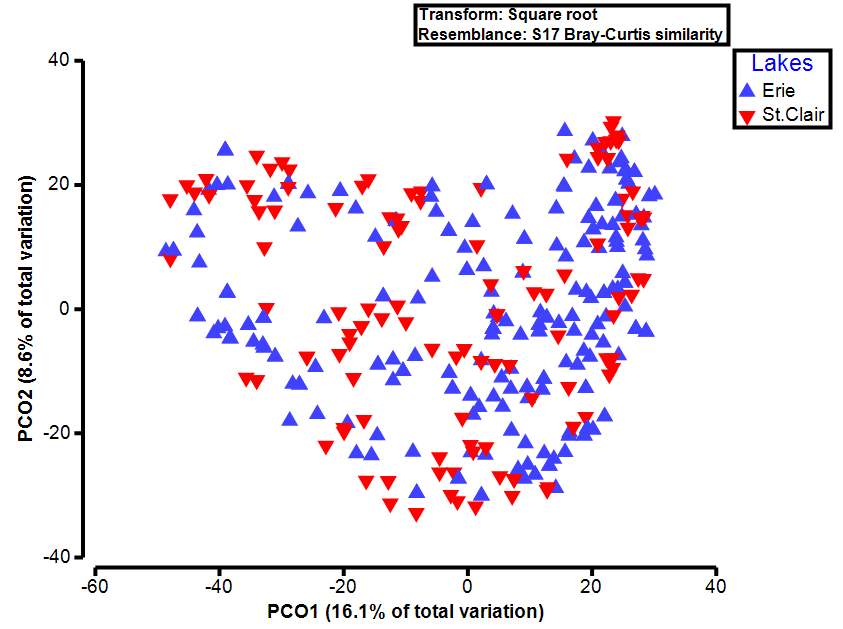


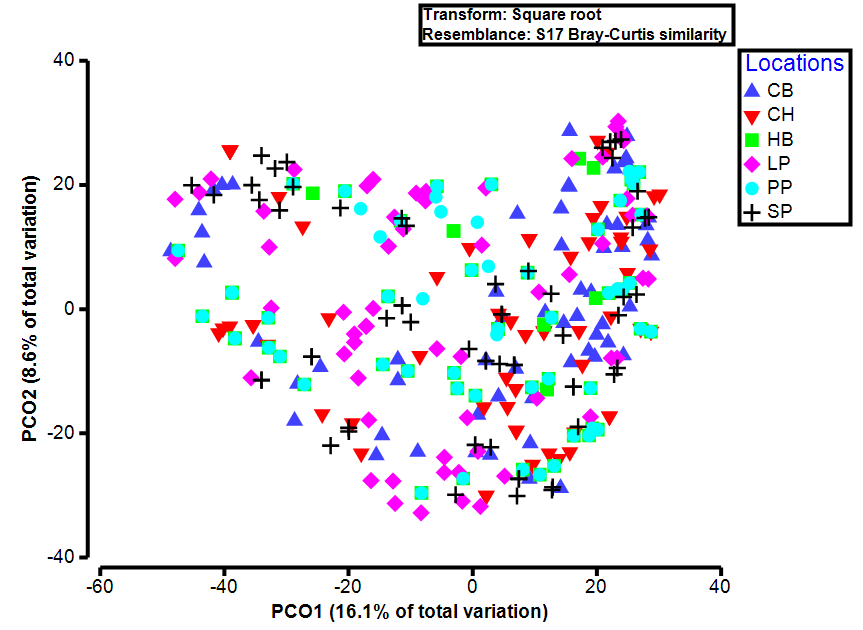


**Supplementary Figure 1.** Multivariant principal coordinate analysis (PCoA) plot of the Bray–Curtis similarity matrix of the BCCs of Lake Erie and Lake St. Clair (Top panel) and six different locations (CB, CH, HB, LP, PP and SP) (bottom panel) across 15 months of sampling.
