# Supplementary material for: Spatio-temporal dynamics of bacterial communities in the shoreline of Laurentian great Lake Erie and Lake St. Clair’s large freshwater ecosystems
Source: BMC Microbiol. 2021 Sep 21;21:253. doi: 10.1186/s12866-021-02306-y (PMC8454060; doi:10.1186/s12866-021-02306-y)
Supplement: Supplementary file 2 — Additional file 2: Supplementary Fig. 2. Taxa with significant spatial variation in their relative abundance among two lakes (Lake Erie and St. Clair). The relative abundance of all 5 classes were significantly higher (p < 0.05) in Lake Eire relative to Lake St. Clair. [file 12866_2021_2306_MOESM2_ESM.docx]

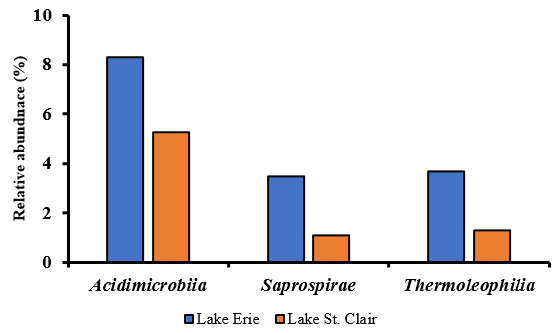

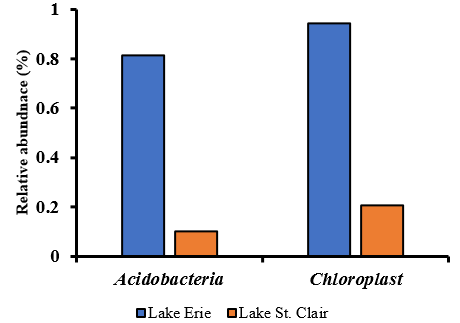


**Supplementary Figure 2.** Taxa with significant spatial variation in their relative abundance among two lakes (Lake Erie and St. Clair). The relative abundance of all 5 classes were significantly higher (p<0.05) in Lake Eire relative to Lake St. Clair.
