# Supplementary material for: Spatio-temporal dynamics of bacterial communities in the shoreline of Laurentian great Lake Erie and Lake St. Clair’s large freshwater ecosystems
Source: BMC Microbiol. 2021 Sep 21;21:253. doi: 10.1186/s12866-021-02306-y (PMC8454060; doi:10.1186/s12866-021-02306-y)
Supplement: Supplementary file 3 — Additional file 3: Supplementary Fig. 3. Bi-weekly variation in the Shannon index for the 6 different sampling locations (CB, CH, HB, LP, PP and SP) over 15 months of sampling (June 2016–August 2017). The X-axis shows time of sampling (bi-weekly sampling). [file 12866_2021_2306_MOESM3_ESM.docx]

**Supplementary Figure 3.** Bi-weekly variation in the Shannon index for the 6 different sampling locations (CB, CH, HB, LP, PP and SP) over 15 months of sampling (June 2016 - August 2017). The X-axis shows time of sampling (bi-weekly sampling).
