# Supplementary material for: Spatio-temporal dynamics of bacterial communities in the shoreline of Laurentian great Lake Erie and Lake St. Clair’s large freshwater ecosystems
Source: BMC Microbiol. 2021 Sep 21;21:253. doi: 10.1186/s12866-021-02306-y (PMC8454060; doi:10.1186/s12866-021-02306-y)
Supplement: Supplementary file 4 — Additional file 4: Supplementary Fig. 4. Line plots of monthly changes of A) Shannon and B) Bray–Curtis dissimilarity components; PCo3–5 of 6 different locations over 15 months of sampling. C1–5: Clusters 1–5 are based on Fig. 4. [file 12866_2021_2306_MOESM4_ESM.docx]

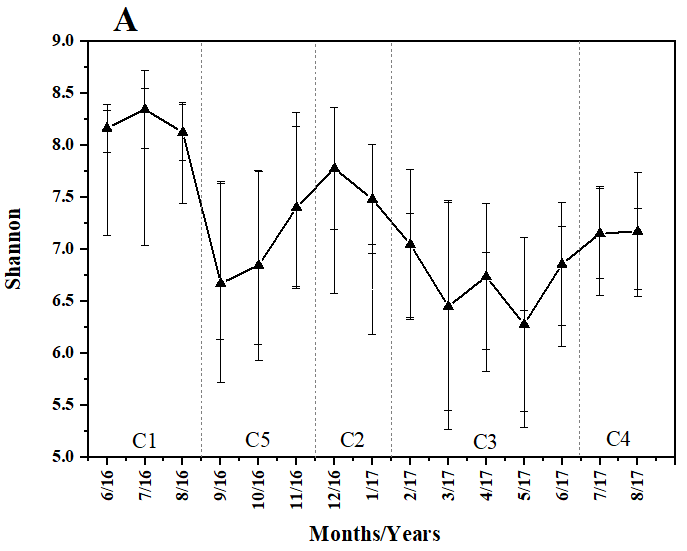


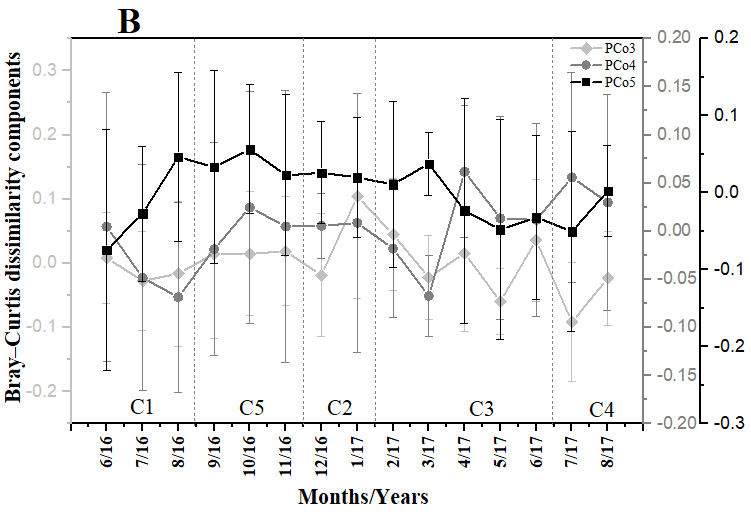


**Supplementary Figure 4.** Line plots of monthly changes of A) Shannon and B) Bray–Curtis dissimilarity components; PCo3-5 of 6 different locations over 15 months of sampling. C1-5: Clusters 1-5 are based on figure 4.
