# Supplementary material for: Spatio-temporal dynamics of bacterial communities in the shoreline of Laurentian great Lake Erie and Lake St. Clair’s large freshwater ecosystems
Source: BMC Microbiol. 2021 Sep 21;21:253. doi: 10.1186/s12866-021-02306-y (PMC8454060; doi:10.1186/s12866-021-02306-y)
Supplement: Supplementary file 5 — Additional file 5: Supplementary Fig. 5. Bar chart showing the relative abundance of the BCCs at the class-level for combined sampling locations and bi-weekly sampling over the 15 months of sampling. [file 12866_2021_2306_MOESM5_ESM.docx]

**Supplementary Figure 5.** Bar chart showing the relative abundance of the BCCs at the class-level for combined sampling locations and bi-weekly sampling over the 15 months of sampling.
