# Supplementary material for: Spatio-temporal dynamics of bacterial communities in the shoreline of Laurentian great Lake Erie and Lake St. Clair’s large freshwater ecosystems
Source: BMC Microbiol. 2021 Sep 21;21:253. doi: 10.1186/s12866-021-02306-y (PMC8454060; doi:10.1186/s12866-021-02306-y)
Supplement: Supplementary file 6 — Additional file 6: Supplementary Fig. 6. Phylogenetic affiliations of the top heterotrophic bacterial (panel a) and phototrophic (panel b) OTU groups from the 15 months of sampling in Lake Erie and St. Clair over 2016 and 2017. Due to space constraints, only taxa that had a relative abundance of more than 1% in at least one sampling month are presented for heterotrophic bacterial (panel a). [file 12866_2021_2306_MOESM6_ESM.docx]

**Supplementary Figure 6.** Phylogenetic affiliations of the top heterotrophic bacterial (panel a) and phototrophic (panel b) OTU groups from the 15 months of sampling in Lake Erie and St. Clair over 2016 and 2017. Due to space constraints, only taxa that had a relative abundance of more than 1% in at least one sampling month are presented for heterotrophic bacterial (panel a).
