# Supplementary material for: Spatio-temporal dynamics of bacterial communities in the shoreline of Laurentian great Lake Erie and Lake St. Clair’s large freshwater ecosystems
Source: BMC Microbiol. 2021 Sep 21;21:253. doi: 10.1186/s12866-021-02306-y (PMC8454060; doi:10.1186/s12866-021-02306-y)
Supplement: Supplementary file 7 — Additional file 7: Supplementary Fig. 7. The pattern of environmental parameter variation (water temperature, precipitation and daylight duration) for the 6 sampling locations over 15 months of the sampling. As air and water temperature both had the same pattern spatially and temporally (no significant variation) we only plotted water temperature. For each month, 2 different weeks were sampled (weeks 1 and 2). Sampling was started in June 2016 and ended in August 2017. Error bars, showing the standard deviation of water temperature and precipitation among 6 sampling locations. [file 12866_2021_2306_MOESM7_ESM.docx]

**Supplementary Figure 7.** The pattern of environmental parameter variation (water temperature, precipitation and daylight duration) for the 6 sampling locations over 15 months of the sampling. As air and water temperature both had the same pattern spatially and temporally (no significant variation) we only plotted water temperature. For each month, 2 different weeks were sampled (weeks 1 and 2). Sampling was started in June 2016 and ended in August 2017. Error bars, showing the standard deviation of water temperature and precipitation among 6 sampling locations.
