# Supplementary material for: Spatio-temporal dynamics of bacterial communities in the shoreline of Laurentian great Lake Erie and Lake St. Clair’s large freshwater ecosystems
Source: BMC Microbiol. 2021 Sep 21;21:253. doi: 10.1186/s12866-021-02306-y (PMC8454060; doi:10.1186/s12866-021-02306-y)
Supplement: Supplementary file 8 — Additional file 8: Supplementary Fig. 8. Distance-based Redundancy Analysis (dbRDA) of freshwater microbiota. The relative position of water samples in the biplot is based on Bray Curtis similarity of arcsine square root transformed relative abundance at the OTU level. Vectors indicate the weight and direction of the environmental variables that were best predictors of the BCCs of different months as suggested by the results of the distance-based linear model (distLM). The dbRDA axes describe the percentage of the fitted or total variation explained by each axis while being constrained to account for group differences. Sample IDs indicate the sampling months. [file 12866_2021_2306_MOESM8_ESM.docx]

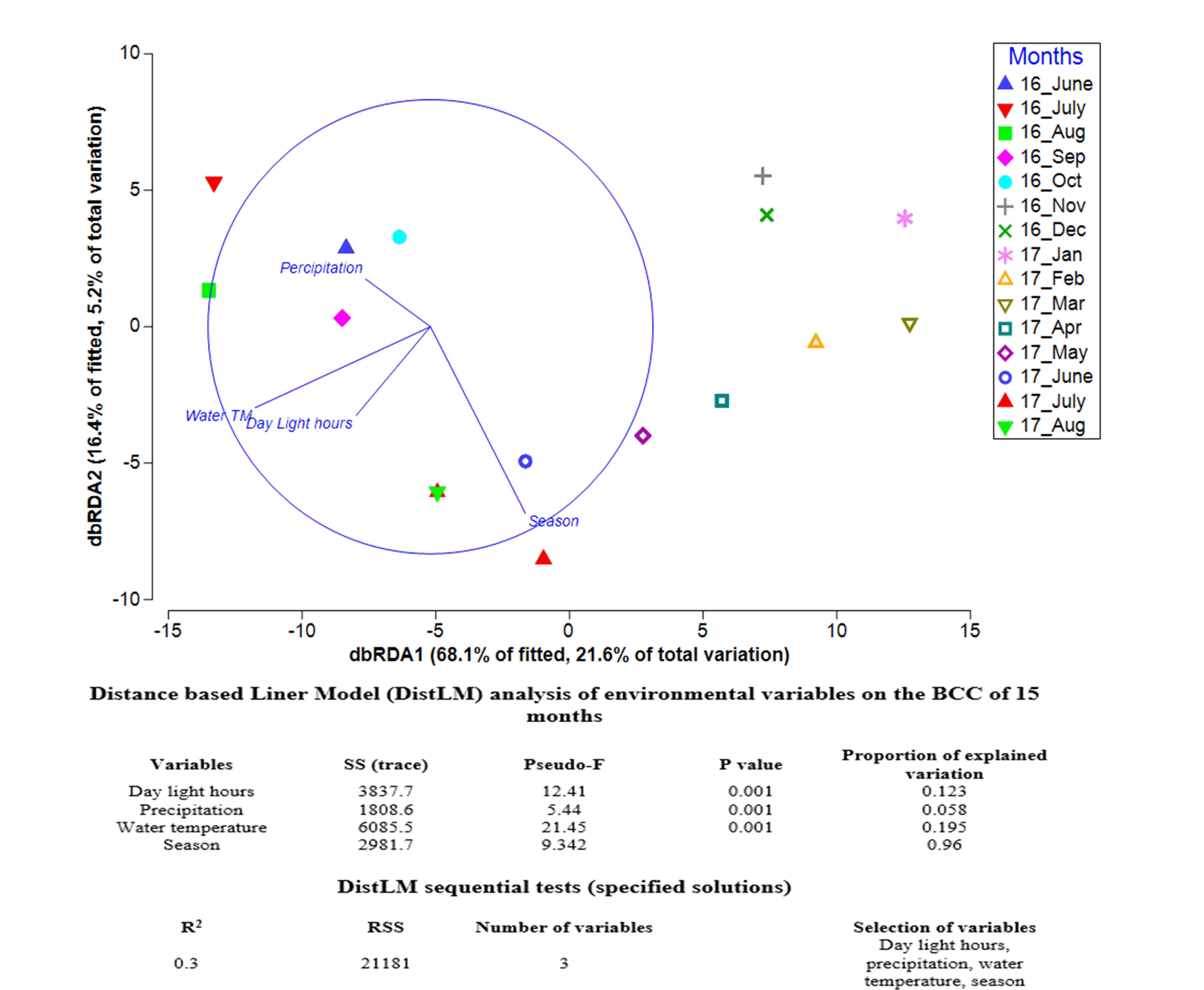


**Supplementary Figure 8.** Distance-based Redundancy Analysis (dbRDA) of freshwater microbiota. The relative position of water samples in the biplot is based on Bray Curtis similarity of square root transformed relative abundance at the OTU level. Vectors indicate the weight and direction of the environmental variables that were best predictors of the BCCs of different months as suggested by the results of the distance-based linear model (distLM). The dbRDA axes describe the percentage of the fitted or total variation explained by each axis while being constrained to account for group differences. Sample IDs indicate the sampling months.
