# Supplementary material for: Spatio-temporal dynamics of bacterial communities in the shoreline of Laurentian great Lake Erie and Lake St. Clair’s large freshwater ecosystems
Source: BMC Microbiol. 2021 Sep 21;21:253. doi: 10.1186/s12866-021-02306-y (PMC8454060; doi:10.1186/s12866-021-02306-y)
Supplement: Supplementary file 9 — Additional file 9: Supplementary Table 1. Results of GLMM analysis of bacterial community variation temporally and spatially. Dependent variables included alpha diversity indexes and Bray–Curtis dissimilarity principal coordinate analysis axes (PCo1 and PCo2). Degrees of freedom, F value and p values are shown (significant p values are highlighted). [file 12866_2021_2306_MOESM9_ESM.docx]

**Supplementary Table 1.** Results of GLMM analysis of bacterial community variation temporally and spatially. Dependent variables included alpha diversity indexes and Bray–Curtis dissimilarity principal coordinate analysis axes (PCo1 and PCo2). Degrees of freedom, F value and p values are shown (significant p values are highlighted).

| **Factors** | **df** | **Chao1** | | **Shannon** | | **PCo1** | | **PCo2** | | **PCo3** | |
| --- | --- | --- | --- | --- | --- | --- | --- | --- | --- | --- | --- |
|  |  | **F value** | **p value** | **F value** | **p value** | **F value** | **P value** | **F value** | **p value** | **F value** | **p value** |
|  | **Full model** | | | | | | | | | | |
| Lake (sampling location) | 5 | 4.8 | **0.000** | 2.7 | **0.02** | 0.002 | 1.000 | 0.000 | 1.000 | 0.000 | 1.000 |
| Month (week) | 29 | 7.11 | **0.000** | 6.9 | **0.000** | 7.4 | **0.000** | 4.8 | **0.000** | 3.8 | **0.000** |
| R^2^ | | 0.4 | | 0.35 | | 0.37 | | 0.27 | |  | |
| **Lake Erie** | | | | | | | | | | | |
| Sampling location | 3 | 6.5 | **0.000** | 5.8 | **0.001** | 0.006 | 0.99 | 0.000 | 1.000 | 0.000 | 1.000 |
| Month | 14 | 21.9 | **0.000** | 24.6 | **0.000** | 21.8 | **0.000** | 61.21 | **0.000** | 55.42 | **0.000** |
| Month (week) | 15 | 4.4 | **0.000** | 5.7 | **0.000** | 4.8 | **0.000** | 3.5 | **0.000** | 3.3 | **0.000** |
| Sampling location x Month (week) | 42 | 3.6 | **0.002** | 3.3 | **0.003** | 2.1 | **0.005** | 2.19 | **0.001** | 2.1 | **0.001** |
| Week | 1 | 0.67 | 0.47 | 1.4 | 0.15 | 0.004 | 0.94 | 1.61 | 0.17 | 1.31 | 0.17 |
| R^2^ | | 0.83 | | 0.79 | | 0.21 | | 0.18 | |  | |
| **Lake St. Clair** | | | | | | | | | | | |
| Sampling location | 1 | 10.3 | **0.000** | 9.3 | **0.000** | 0.003 | 0.95 | 0.000 | 1.000 | 0.000 | 1.000 |
| Month | 14 | 24.2 | **0.000** | 14.8 | **0.000** | 48.8 | **0.000** | 25.2 | **0.000** | 23.2 | **0.000** |
| Month (week) | 14 | 2.5 | **0.001** | 2.4 | **0.003** | 12.4 | **0.000** | 9.1 | **0.000** | 8.3 | **0.000** |
| Sampling location x Month (week) | 14 | 2.1 | **0.01** | 7.1 | **0.000** | 6.1 | **0.000** | 5.9 | **0.000** | 4.3 | **0.000** |
| Week | 1 | 0.18 | 0.66 | 1.1 | 0.35 | 0.23 | 0.62 | 1.56 | 0.25 | 1.22 | 0.25 |
| R^2^ | | 0.29 | | 0.26 | | 0.2 | | 0.12 | |  | |
| Sampling location; 6 public beaches, Month; 15 sampling months, Week; bi-weekly sampling/month, Lake; two lakes. Parenthesis indicates nesting the variables and “x” indicates interaction effects. | | | | | | | | | | | |
