# Supplementary material for: Spatio-temporal dynamics of bacterial communities in the shoreline of Laurentian great Lake Erie and Lake St. Clair’s large freshwater ecosystems
Source: BMC Microbiol. 2021 Sep 21;21:253. doi: 10.1186/s12866-021-02306-y (PMC8454060; doi:10.1186/s12866-021-02306-y)
Supplement: Supplementary file 10 — Additional file 10: Supplementary Table 2. List of top 20 abundant OTUs affected by time, location and their interaction. [file 12866_2021_2306_MOESM10_ESM.docx]

**Supplementary Table 2.** List of top 20 abundant OTUs affected by time, location, and their interaction.

| **OUT IDs** | **Phyla** | **Class** | **Order** | **Family** | **Affected by** |
| --- | --- | --- | --- | --- | --- |
| OTU2 | *Actinobacteria* | *Actinobacteria* | *Actinomycetales* | ACK-M1 | Time |
| OTU3 | *Actinobacteria* | *Actinobacteria* | *Actinomycetales* | ACK-M1 | Time |
| OTU4 | *Firmicutes* | *Bacilli* | *Bacillales* | *Exiguobacteraceae* | Time |
| OTU6 | *Proteobacteria* | *Betaproteobacteria* | *Burkholderiales* | *Comamonadaceae* | Time |
| OTU9 | *Proteobacteria* | *Betaproteobacteria* | *Burkholderiales* | *Oxalobacteraceae* | Time |
| OTU10 | *Actinobacteria* | *Actinobacteria* | *Actinomycetales* | - | Time |
| OTU14 | *Firmicutes* | *Bacilli* | *Bacillales* | *Bacillaceae* | Time |
| OTU8 | *Actinobacteria* | *Actinobacteria* | *Actinomycetales* | ACK-M1 | Time |
| OTU7 | *Actinobacteria* | *Acidimicrobiia* | *Acidimicrobiales* | C111 | Time |
| OTU15 | *Proteobacteria* | *Betaproteobacteria* | *Burkholderiales* | *Comamonadaceae* | Time |
| OTU19 | *Proteobacteria* | *Betaproteobacteria* | *Burkholderiales* | *Comamonadaceae* | Time |
| OTU13 | *Bacteroidetes* | *Cytophagia* | *Cytophagales* | *Cyclobacteriaceae* | Time |
| OTU12 | *Firmicutes* | *Bacilli* | *Bacillales* | *Bacillaceae* | Time |
| OTU17 | *Proteobacteria* | *Betaproteobacteria* | *Methylophilales* | *Methylophilaceae* | Time |
| OTU11 | *Actinobacteria* | *Actinobacteria* | *Actinomycetales* | *-* | Time |
| OTU26 | *Firmicutes* | *Bacilli* | *Bacillales* | *Bacillaceae* | Time |
| OTU5 | *Proteobacteria* | *Gammaproteobacteria* | *Enterobacteriales* | *Enterobacteriaceae* | Time |
| OTU30 | *Proteobacteria* | *Betaproteobacteria* | *-* | - | Time |
| OTU21 | *Proteobacteria* | *Gammaproteobacteria* | *Enterobacteriales* | *Enterobacteriaceae* | Time |
| OTU18 | *Bacteroidetes* | *Sphingobacteriia* | *Sphingobacteriales* | - | Time |
| OTU240 | *Actinobacteria* | *Acidimicrobiia* | *Acidimicrobiales* | C111 | Location |
| OTU282 | *Bacteroidetes* | *Saprospirae* | *Saprospirales* | *Chitinophagaceae* | Location |
| OTU216 | *Actinobacteria* | *Actinobacteria* | *Actinomycetales* | ACK-M1 | Location |
| OTU243 | *Actinobacteria* | *Actinobacteria* | *Actinomycetales* | ACK-M1 | Location |
| OTU437 | *Proteobacteria* | *Alphaproteobacteria* | *Sphingomonadales* | *Sphingomonadaceae* | Location |
| OTU645 | *Actinobacteria* | *Actinobacteria* | *Actinomycetales* | *Microbacteriaceae* | Location |
| OTU322 | *Firmicutes* | *Bacilli* | *Bacillales* | *Exiguobacteraceae* | Location |
| OTU605 | *Actinobacteria* | *Actinobacteria* | *Actinomycetales* | *Microbacteriaceae* | Location |
| OTU532 | *Firmicutes* | *Bacilli* | *Bacillales* | - | Location |
| OTU214 | *Actinobacteria* | *Actinobacteria* | *Actinomycetales* | ACK-M1 | Location |
| OTU454 | *Cyanobacteria* | *Chloroplast* | *Stramenopiles* | - | Location |
| OTU636 | *Proteobacteria* | *Gammaproteobacteria* | *Enterobacteriales* | *Enterobacteriaceae* | Location |
| OTU306 | *Firmicutes* | *Bacilli* | *Bacillales* | *Bacillaceae* | Location |
| OTU250 | *Gemmatimonadetes* | *Gemmatimonadetes* | *KD8-87* | *-* | Location |
| OTU496 | *Proteobacteria* | *Betaproteobacteria* | *Burkholderiales* | *Oxalobacteraceae* | Location |
| OTU387 | *Bacteroidetes* | *Flavobacteriia* | *Flavobacteriales* | *Cryomorphaceae* | Location |
| OTU307 | *Bacteroidetes* | *Sphingobacteriia* | *Sphingobacteriales* | *-* | Location |
| OTU598 | *Proteobacteria* | *Gammaproteobacteria* | *Enterobacteriales* | *Enterobacteriaceae* | Location |
| OTU217 | *Actinobacteria* | *Actinobacteria* | *Actinomycetales* | *-* | Location |
| OTU293 | *Firmicutes* | *Bacilli* | *Bacillales* | *Paenibacillaceae* | Location |
| OTU626 | *Proteobacteria* | *Betaproteobacteria* | *Burkholderiales* | *Comamonadaceae* | Time*Location |
| OTU302 | *Bacteroidetes* | *Sphingobacteriia* | *Sphingobacteriales* | *-* | Time*Location |
| OTU622 | *Proteobacteria* | *Alphaproteobacteria* | *Rhizobiales* | *Bradyrhizobiaceae* | Time*Location |
| OTU360 | *Proteobacteria* | *Alphaproteobacteria* | *-* | *-* | Time*Location |
| OTU272 | *Verrucomicrobia* | *Verrucomicrobiae* | *Verrucomicrobiales* | *Verrucomicrobiaceae* | Time*Location |
| OTU466 | *Proteobacteria* | *Betaproteobacteria* | *Burkholderiales* | *Comamonadaceae* | Time*Location |
| OTU756 | *Firmicutes* | *Bacilli* | *Bacillales* | *Exiguobacteraceae* | Time*Location |
| OTU842 | *Firmicutes* | *Bacilli* | *Bacillales* | *Bacillaceae* | Time*Location |
| OTU413 | *Proteobacteria* | *Betaproteobacteria* | *Rhodocyclales* | *Rhodocyclaceae* | Time*Location |
| OTU181 | *Proteobacteria* | *Alphaproteobacteria* | *Rhizobiales* | *-* | Time*Location |
| OTU584 | *Proteobacteria* | *Gammaproteobacteria* | *Pseudomonadales* | *Pseudomonadaceae* | Time*Location |
| OTU732 | *Bacteroidetes* | *Flavobacteriia* | *Flavobacteriales* | *Flavobacteriaceae* | Time*Location |
| OTU607 | *Proteobacteria* | *Alphaproteobacteria* | *Sphingomonadales* | *Sphingomonadaceae* | Time*Location |
| OTU313 | *Firmicutes* | *Bacilli* | *Bacillales* | *Exiguobacteraceae* | Time*Location |
| OTU346 | *Proteobacteria* | *Alphaproteobacteria* | *Rhizobiales* | - | Time*Location |
| OTU427 | *Actinobacteria* | *Actinobacteria* | *Actinomycetales* | ACK-M1 | Time*Location |
| OTU342 | *Proteobacteria* | *Betaproteobacteria* | *Burkholderiales* | *Oxalobacteraceae* | Time*Location |
| OTU799 | *Firmicutes* | *Bacilli* | *Bacillales* | *Planococcaceae* | Time*Location |
| OTU327 | *Firmicutes* | *Bacilli* | *Bacillales* | *Exiguobacteraceae* | Time*Location |
| OTU965 | *Firmicutes* | *Bacilli* | *Bacillales* | *Bacillaceae* | Time*Location |
