# Supplementary material for: Spatio-temporal dynamics of bacterial communities in the shoreline of Laurentian great Lake Erie and Lake St. Clair’s large freshwater ecosystems
Source: BMC Microbiol. 2021 Sep 21;21:253. doi: 10.1186/s12866-021-02306-y (PMC8454060; doi:10.1186/s12866-021-02306-y)
Supplement: Supplementary file 11 — Additional file 11: Supplementary Table 3. Taxa which shown significant spatial variation (p < 0.05) in the six sampling locations in Lakes Erie and St Clair. [file 12866_2021_2306_MOESM11_ESM.docx]

**Supplementary Table 3.** Taxa which shown significant spatial variation (p<0.05) in the six sampling locations in Lakes Erie and St Clair.

| **Phylum.Class.Order.Family*** | **Locations (Pairwise)** | **LDA effect** | **p value** |
| --- | --- | --- | --- |
|  | **CB-CH** |  |  |
| *Firmicutes.Bacilli.Bacillales* | CH | 3.43 | 0.002 |
| *Gemmatimonadetes.Gemmatimonadetes.KD8-87* | CB | 2.36 | 0.003 |
| *Proteobacteria.Gammaproteobacteria,* *Alteromonadales* | CB | 3.0 | 0.001 |
| *Verrucomicrobia.Verrucomicrobiae. Verrucomicrobiales.Verrucomicrobiaceae* | CB | 2.4 | 0.026 |
|  | **CB-HB** |  |  |
| *Acidobacteria.Solibacteres.Solibacterales. Bryobacteraceae* | CB | 2.55 | 0.037 |
| *Cyanobacteria.Chloroplast.Chlorophyta.Chlamydomonadaceae* | CB | 2.54 | 0.047 |
| *Cyanobacteria.Nostocophycideae.Nostocales.Nostocaceae* | CB | 2.17 | 0.016 |
| *Firmicutes.Bacilli.Bacillales* | HB | 3.23 | 0.001 |
|  | **CB-LP** |  |  |
| *Actinobacteria.Acidimicrobiia.* *Acidimicrobiales* | CB | 3.77 | 0.023 |
| *Actinobacteria.Thermoleophilia.* *Gaiellales.Gaiellaceae* | CB | 3.42 | 0.029 |
| *Bacteroidetes.Saprospirae.* *Saprospirales.Chitinophagaceae* | CB | 3.48 | 0.033 |
| *Cyanobacteria.Chloroplast.Chlorophyta.Chlamydomonadaceae* | CB | 3.2 | 0.011 |
| *Firmicutes.Bacilli.Bacillales* | LP | 3.11 | 0.001 |
|  | **CB-PP** |  |  |
| *Cyanobacteria.Chloroplast.Chlorophyta.Chlamydomonadaceae* | CB | 3.32 | 0.028 |
| *Firmicutes.Bacilli.Bacillales* | PP | 3.33 | 0.003 |
|  | **CB-SP** |  |  |
| *Actinobacteria.Acidimicrobiia.* *Acidimicrobiales* | CB | 3.39 | 0.002 |
| *Bacteroidetes.Flavobacteriia.Flavobacteriales.Flavobacteriaceae* | CB | 3.05 | 0.012 |
| *Bacteroidetes.Saprospirae.* *Saprospirales.Chitinophagaceae* | CB | 3.47 | 0.010 |
| *Cyanobacteria.Chloroplast.* *Chlorophyta.Trebouxiophyceae* | CB | 2.52 | 0.024 |
| *Firmicutes.Bacilli.Bacillales* | CB | 2.43 | 0.001 |
|  | **CH-HB** |  |  |
| *Cyanobacteria.Chloroplast* | CH | 3.07 | 0.032 |
|  | **CH-LP** |  |  |
| *Actinobacteria.Acidimicrobiia.* *Acidimicrobiales* | CH | 3.85 | 0.006 |
| *Planctomycetes.Planctomycetia.Pirellulales.Pirellulaceae* | LP | 4.12 | 0.014 |
| *Proteobacteria.Gammaproteobacteria. Aeromonadales* | LP | 4.01 | 0.024 |
|  | **CH-SP** |  |  |
| *Actinobacteria.Acidimicrobiia.* *Acidimicrobiales* | CH | 3.75 | 0.005 |
| *Actinobacteria.Thermoleophilia.* *Gaiellales.Gaiellaceae* | CH | 3.29 | 0.047 |
| *Bacteroidetes.Flavobacteriia.Flavobacteriales.Cryomorphaceae* | CH | 3.37 | 0.008 |
| *Cyanobacteria.Chloroplast* | CH | 3.14 | 0.049 |
| *Proteobacteria.Gammaproteobacteria.* *Aeromonadales* | SP | 3.54 | 0.003 |
|  | **HB-LP** |  |  |
| *Bacteroidetes.Sphingobacteriia.Sphingobacteriales.Sphingobacteriaceae* | LP | 3.32 | 0.026 |
| *Proteobacteria.Gammaproteobacteria.* *Aeromonadales* | LP | 2.45 | 0.003 |
|  | **HB-SP** |  |  |
| *Acidobacteria.Acidobacteria 6.* *CCU21* | HB | 4.01 | 0.009 |
| *Bacteroidetes.Flavobacteriia.Flavobacteriales.Cryomorphaceae* | HB | 3.57 | 0.004 |
| *Gemmatimonadetes.Gemmatimonadetes.KD8-87* | HB | 2.61 | 0.042 |
| *Proteobacteria.Gammaproteobacteria.* *Aeromonadales* | SP | 3.45 | 0.003 |
| *Verrucomicrobia.Pedosphaerae. Pedosphaerales.R4-41B* | HB | 2.4 | 0.030 |
|  | **PP-LP** |  |  |
| *Actinobacteria.Acidimicrobiia.* *Acidimicrobiales* | PP | 4.0 | 0.020 |
| *Proteobacteria.Gammaproteobacteria.* *Aeromonadales* | LP | 2.43 | 0.002 |
|  | **PP-SP** |  |  |
| *Acidobacteria.Acidobacteria 6.* *CCU21* | PP | 3.87 | 0.025 |
| *Actinobacteria.Acidimicrobiia.* *Acidimicrobiales* | PP | 3.70 | 0.017 |
| *Bacteroidetes.Flavobacteriia.Flavobacteriales.Cryomorphaceae* | PP | 3.66 | 0.006 |
| *Proteobacteria.Gammaproteobacteria.* *Aeromonadales* | SP | 2.22 | 0.004 |
|  | **LP-SP** |  |  |
| *Bacteroidetes.Bacteroidia.Bacteroidales.Porphyromonadaceae* | LP | 2.13 | 0.015 |
| *Bacteroidetes.Flavobacteriia.Flavobacteriales.Flavobacteriaceae* | LP | 3.63 | 0.018 |
| *Planctomycetes.Phycisphaerae.* *Phycisphaerales* | LP | 2.14 | 0.013 |
| *Planctomycetes.Planctomycetia.Pirellulales.Pirellulaceae* | LP | 3.04 | 0.011 |
| *Verrucomicrobia.Verrucomicrobiae.Verrucomicrobiales.Verrucomicrobiaceae* | LP | 2.26 | 0.037 |
| *All bacteria did not have taxa information at family level. | | | |
