# Supplementary material for: Spatio-temporal dynamics of bacterial communities in the shoreline of Laurentian great Lake Erie and Lake St. Clair’s large freshwater ecosystems
Source: BMC Microbiol. 2021 Sep 21;21:253. doi: 10.1186/s12866-021-02306-y (PMC8454060; doi:10.1186/s12866-021-02306-y)
Supplement: Supplementary file 12 — Additional file 12: Supplementary Table 4. SIMPER results (above the diagonal) and pairwise PERMANOVA probabilities (below the diagonal) of 5 broad clusters of the BCCs. p values were adjusted using a sequential Bonferroni correction for multiple comparisons. [file 12866_2021_2306_MOESM12_ESM.docx]

**Supplementary Table 4.** SIMPER results (above the diagonal) and pairwise PERMANOVA probabilities (below the diagonal) of 5 broad clusters of the BCCs. p values were adjusted using a sequential Bonferroni correction for multiple comparisons.

| **Clusters** | **1** | **2** | **3** | **4** | **5** |
| --- | --- | --- | --- | --- | --- |
| **1** |  | 54.12 | 55.75 | 49.61 | 47 |
| **2** | **0.0001** |  | 50.21 | 57.74 | 48.12 |
| **3** | **0.0001** | **0.0001** |  | 51.72 | 49.55 |
| **4** | **0.0001** | **0.0001** | **0.0001** |  | 49.36 |
| **5** | **0.0001** | **0.0001** | **0.0001** | **0.0001** |  |
