# Supplementary material for: Spatio-temporal dynamics of bacterial communities in the shoreline of Laurentian great Lake Erie and Lake St. Clair’s large freshwater ecosystems
Source: BMC Microbiol. 2021 Sep 21;21:253. doi: 10.1186/s12866-021-02306-y (PMC8454060; doi:10.1186/s12866-021-02306-y)
Supplement: Supplementary file 13 — Additional file 13: Supplementary Table 5. Pairwise comparison of diversity indexes between the BCCs of 5 clusters. [file 12866_2021_2306_MOESM13_ESM.docx]

**Supplementary Table 5.** Pairwise comparison of diversity indexes between the BCCs of 5 clusters.

| **Clusters** | | **Chao1** | **Shannon** | **PCo1** | **PCo2** |
| --- | --- | --- | --- | --- | --- |
|  |  |  |  |  |  |
| **1** | **2** | **0.000** | **0.014** | **0.000** | **0.000** |
|  | **3** | **0.000** | **0.000** | **0.000** | **0.000** |
|  | **4** | **0.000** | **0.000** | **0.009** | 0.881 |
|  | **5** | **0.000** | **0.000** | **0.000** | 0.776 |
| **2** | **1** | **0.000** | **0.014** | **0.000** | **0.000** |
|  | **3** | **0.003** | **0.000** | **0.017** | **0.001** |
|  | **4** | 0.995 | 0.092 | 0.4 | **0.000** |
|  | **5** | 0.998 | **0.004** | 0.83 | **0.000** |
| **3** | **1** | **0.000** | **0.000** | **0.000** | **0.000** |
|  | **2** | **0.003** | **0.000** | **0.017** | **0.001** |
|  | **4** | **0.016** | 0.053 | **0.000** | **0.000** |
|  | **5** | **0.000** | 0.239 | 0.156 | **0.000** |
| **4** | **1** | **0.000** | **0.000** | **0.009** | 0.881 |
|  | **2** | 0.995 | 0.092 | 0.400 | **0.000** |
|  | **3** | **0.016** | **0.053** | **0.000** | **0.000** |
|  | **5** | 0.946 | 0.914 | **0.027** | 0.283 |
| **5** | **1** | **0.000** | **0.000** | **0.000** | 0.776 |
|  | **2** | 0.998 | **0.004** | 0.833 | **0.000** |
|  | **3** | **0.000** | 0.239 | 0.156 | **0.000** |
|  | **4** | 0.946 | 0.914 | **0.027** | 0.283 |
