# Supplementary material for: Spatio-temporal dynamics of bacterial communities in the shoreline of Laurentian great Lake Erie and Lake St. Clair’s large freshwater ecosystems
Source: BMC Microbiol. 2021 Sep 21;21:253. doi: 10.1186/s12866-021-02306-y (PMC8454060; doi:10.1186/s12866-021-02306-y)
Supplement: Supplementary file 14 — Additional file 14: Supplementary Table 6. Pairwise comparison of temporal variation of taxa (class level) between the BCCs of 5 clusters. [file 12866_2021_2306_MOESM14_ESM.docx]

**Supplementary Table 6.** Pairwise comparison of temporal variation of taxa (class level) between the BCCs of 5 clusters.

| Phylum.Class | Clusters | LDA effect | p value |
| --- | --- | --- | --- |
| Cluster 1 versus Cluster-2 | | | |
| *Acidobacteria.Solibacteres* | 1 | 4.042 | **0.0025** |
| *Actinobacteria.Acidimicrobiia* | 1 | 4.180 | **0.0022** |
| *Actinobacteria.Actinobacteria* | 1 | 4.650 | **0.0026** |
| *Actinobacteria.Thermoleophilia* | 1 | 3.763 | **0.002** |
| *Bacteroidetes.Cytophagia* | 1 | 3.861 | **0.0028** |
| *Bacteroidetes.Saprospirae* | 1 | 3.867 | **0.0024** |
| *Cyanobacteria.Chloroplast* | 3 | 3.399 | **0.0021** |
| *Cyanobacteria.Synechococcophycideae* | 3 | 3.583 | **0.0018** |
| *Firmicutes. Bacilli* | 3 | 4.457 | **0.0023** |
| *Gemmatimonadetes.Gemmatimonadetes* | 1 | 3.641 | **0.0025** |
| *Planctomycetes.Phycisphaerae* | 1 | 3.282 | **0.002** |
| *Planctomycetes.Planctomycetia* | 1 | 3.148 | **0.0024** |
| *Proteobacteria.Betaproteobacteria* | 3 | 4.088 | **0.0027** |
| *Proteobacteria.Gammaproteobacteria* | 3 | 4.204 | **0.0024** |
| *Verrucomicrobia.Pedosphaerae* | 1 | 3.201 | **0.0025** |
| Cluster 1 versus Cluster 3 | | | |
| *Acidobacteria.Acidobacteria_6* | 1 | 3.028 | **0.0018** |
| *Acidobacteria.Solibacteres* | 1 | 3.704 | **0.0023** |
| *Actinobacteria.Acidimicrobiia* | 1 | 4.072 | **0.0025** |
| *Actinobacteria.Actinobacteria* | 1 | 4.674 | **0.002** |
| *Actinobacteria.Thermoleophilia* | 1 | 3.740 | **0.0024** |
| *Bacteroidetes.Cytophagia* | 1 | 3.856 | **0.0027** |
| *Bacteroidetes.Saprospirae* | 1 | 3.822 | **0.0018** |
| *Chloroflexi.Chloroflexi* | 1 | 2.895 | **0.0023** |
| *Cyanobacteria.Chloroplast* | 4 | 2.829 | **0.0025** |
| *Cyanobacteria.Nostocophycideae* | 1 | 3.313 | **0.0015** |
| *Firmicutes.Bacilli* | 4 | 4.830 | **0.0025** |
| *Gemmatimonadetes.Gemmatimonadetes* | 1 | 3.065 | **0.0095** |
| *Planctomycetes.Phycisphaerae* | 1 | 2.845 | **0.007** |
| *Proteobacteria.Gammaproteobacteria* | 4 | 4.138 | **0.006** |
| *Verrucomicrobia.Pedosphaerae* | 1 | 2.928 | **0.008** |
| *Verrucomicrobia.Verrucomicrobiae* | 1 | 2.575 | **0.002** |
| Cluster 1 versus Cluster 4 | | | |
| *Acidobacteria.Acidobacteria_6* | 1 | 2.869 | **0.0025** |
| *Acidobacteria.Solibacteres* | 1 | 3.359 | **0.0022** |
| *Actinobacteria.Actinobacteria* | 1 | 4.573 | **0.0026** |
| *Actinobacteria.Thermoleophilia* | 1 | 3.534 | **0.002** |
| *Bacteroidetes.Cytophagia* | 1 | 3.894 | **0.0028** |
| *Bacteroidetes.Flavobacteriia* | 1 | 3.566 | **0.0024** |
| *Bacteroidetes.Saprospirae* | 1 | 3.745 | **0.0018** |
| *Bacteroidetes.Sphingobacteriia* | 1 | 2.889 | **0.0023** |
| *Chloroflexi.Chloroflexi* | 1 | 2.665 | **0.0025** |
| *Cyanobacteria.Nostocophycideae* | 1 | 3.066 | **0.002** |
| *Cyanobacteria.Synechococcophycideae* | 5 | 2.784 | **0.0024** |
| *Firmicutes.Bacilli* | 5 | 4.773 | **0.0027** |
| *Firmicutes.Clostridia* | 1 | 3.914 | **0.0018** |
| *Gemmatimonadetes.Gemmatimonadetes* | 1 | 2.742 | **0.0023** |
| *Nitrospirae.Nitrospira* | 1 | 3.637 | **0.0025** |
| *Planctomycetes.Phycisphaerae* | 1 | 3.008 | **0.0015** |
| *Proteobacteria.Alphaproteobacteria* | 1 | 3.269 | **0.0025** |
| *Proteobacteria.Betaproteobacteria* | 1 | 4.056 | **0.0095** |
| *Proteobacteria.Deltaproteobacteria* | 1 | 3.121 | **0.0018** |
| *Thermi.Deinococci* | 1 | 2.216 | **0.0023** |
| *Verrucomicrobia.Pedosphaerae* | 1 | 2.844 | **0.0046** |
| *Verrucomicrobia.Verrucomicrobiae* | 1 | 2.494 | **0.0034** |
| Cluster 1 versus Cluster 5 | | | |
| *Actinobacteria.Acidimicrobiia* | 1 | 4.061 | **0.0025** |
| *Actinobacteria.Actinobacteria* | 1 | 4.561 | **0.002** |
| *Actinobacteria.Thermoleophilia* | 1 | 3.732 | **0.0024** |
| *Bacteroidetes.Cytophagia* | 1 | 3.894 | **0.0027** |
| *Bacteroidetes.Flavobacteriia* | 1 | 3.671 | **0.0018** |
| *Bacteroidetes.Saprospirae* | 1 | 3.828 | **0.0023** |
| *Bacteroidetes.Sphingobacteriia* | 1 | 4.077 | **0.0025** |
| *Firmicutes.Bacilli* | 2 | 4.743 | **0.0052** |
| *Planctomycetes.Phycisphaerae* | 1 | 3.674 | **0.0032** |
| *Verrucomicrobia.Pedosphaerae* | 1 | 3.742 | **0.002** |
| Cluster 2 versus Cluster 3 | | | |
| *Acidobacteria.Solibacteres* | 3 | 3.229 | **0.004** |
| *Chloroflexi.Chloroflexi* | 3 | 2.668 | **0.0094** |
| *Cyanobacteria.Chloroplast* | 3 | 2.671 | **0.002** |
| *Cyanobacteria.Nostocophycideae* | 3 | 3.041 | **0.0052** |
| *Cyanobacteria.Synechococcophycideae* | 3 | 3.500 | **0.0032** |
| *Firmicutes.Bacilli* | 4 | 4.615 | **0.002** |
| *Planctomycetes.Phycisphaerae* | 3 | 2.771 | **0.005** |
| *Proteobacteria* | 3 | 4.480 | **0.008** |
| Cluster 2 versus Cluster 4 | | | |
| *Acidobacteria.Acidobacteria* | 3 | 2.319 | **0.0085** |
| *Acidobacteria.Solibacteres* | 3 | 2.438 | **0.0072** |
| *Actinobacteria.Acidimicrobiia* | 5 | 3.992 | **0.008** |
| *Actinobacteria.Thermoleophilia* | 5 | 3.128 | **0.007** |
| *Bacteroidetes.Flavobacteriia* | 3 | 3.866 | **0.0028** |
| *Bacteroidetes.Saprospirae* | 5 | 3.314 | **0.0024** |
| *Bacteroidetes.Sphingobacteriia* | 3 | 3.494 | **0.0032** |
| *Chloroflexi.Chloroflexi* | 3 | 2.565 | **0.0018** |
| *Cyanobacteria.Chloroplast* | 3 | 2.936 | **0.0023** |
| *Cyanobacteria.Nostocophycideae* | 3 | 3.102 | **0.0025** |
| *Cyanobacteria.Synechococcophycideae* | 3 | 3.430 | **0.002** |
| *Firmicutes.Bacilli* | 5 | 4.459 | **0.0024** |
| *Firmicutes.Clostridia* | 3 | 2.612 | **0.0027** |
| *Nitrospirae.Nitrospira* | 3 | 2.733 | **0.0024** |
| *Planctomycetes.Planctomycetia* | 5 | 3.449 | **0.0025** |
| *Proteobacteria.Alphaproteobacteria* | 3 | 3.564 | **0.0022** |
| *Proteobacteria.Betaproteobacteria* | 3 | 4.323 | **0.0026** |
| *Proteobacteria.Deltaproteobacteria* | 3 | 4.078 | **0.002** |
| Cluster 2 versus Cluster 5 | | | |
| *Actinobacteria.Acidimicrobiia* | 2 | 3.778 | **0.0028** |
| *Bacteroidetes.Flavobacteriia* | 3 | 3.891 | **0.0024** |
| *Bacteroidetes.Saprospirae* | 2 | 3.565 | **0.0032** |
| *Cyanobacteria.Nostocophycideae* | 2 | 4.261 | **0.0018** |
| *Cyanobacteria.Synechococcophycideae* | 3 | 3.630 | **0.0023** |
| *Firmicutes.Bacilli* | 2 | 4.517 | **0.0025** |
| *Planctomycetes.Planctomycetia* | 2 | 3.425 | **0.002** |
| *Proteobacteria.Betaproteobacteria* | 3 | 4.251 | **0.0024** |
| *Verrucomicrobia.Pedosphaerae* | 2 | 3.795 | **0.0028** |
| Cluster 3 versus Cluster 4 | | | |
| *Actinobacteria.Acidimicrobiia* | 5 | 3.935 | **0.0034** |
| *Actinobacteria.Actinobacteria* | 5 | 4.115 | **0.0024** |
| *Actinobacteria.Thermoleophilia* | 5 | 3.473 | **0.009** |
| *Bacteroidetes.Saprospirae* | 5 | 3.569 | **0.007** |
| *Cyanobacteria.Chloroplast* | 4 | 3.842 | **0.0043** |
| *Cyanobacteria.Synechococcophycideae* | 5 | 3.663 | **0.0053** |
| *Planctomycetes.Planctomycetia* | 5 | 3.648 | **0.0097** |
| Cluster 3 versus Cluster 5 | | | |
| *Actinobacteria.Actinobacteria* | 2 | 4.208 | **0.0023** |
| *Cyanobacteria.Nostocophycideae* | 2 | 4.016 | **0.0025** |
| *Proteobacteria* | 4 | 4.087 | **0.002** |
| *Verrucomicrobia.Pedosphaerae* | 2 | 3.612 | **0.0024** |
| Cluster 4 versus Cluster 5 | | | |
| *Actinobacteria.Acidimicrobiia* | 5 | 3.708 | **0.002** |
| *Cyanobacteria.Nostocophycideae* | 2 | 2.522 | **0.0052** |
| *Proteobacteria.Alphaproteobacteria* | 2 | 3.269 | **0.0032** |
| *Proteobacteria.Betaproteobacteria* | 2 | 3.804 | **0.002** |
| *Proteobacteria.Deltaproteobacteria* | 2 | 3.020 | **0.005** |
| *Verrucomicrobia.Pedosphaerae* | 2 | 2.458 | **0.002** |
