# Supplementary material for: Spatio-temporal dynamics of bacterial communities in the shoreline of Laurentian great Lake Erie and Lake St. Clair’s large freshwater ecosystems
Source: BMC Microbiol. 2021 Sep 21;21:253. doi: 10.1186/s12866-021-02306-y (PMC8454060; doi:10.1186/s12866-021-02306-y)
Supplement: Supplementary file 15 — Additional file 15: Supplementary Table 7. Pairwise dissimilarity (%; SIMPER) (above the diagonal) and PERMANOVA significance probabilities (below the diagonal) for the BCCs across the 15 months of sampling (numbers indicate months, 16 and 17 show 2016 and 2017 respectively. P values were adjusted using a Bonferroni correction for multiple comparisons. [file 12866_2021_2306_MOESM15_ESM.docx]

**Supplementary Table 7.** Pairwise dissimilarity (%; SIMPER) (above the diagonal) and PERMANOVA significance probabilities (below the diagonal) for the BCCs across the 15 months of sampling (numbers indicate months, 16 and 17 show 2016 and 2017 respectively. P values were adjusted using a Bonferroni correction for multiple comparisons.

| **Month**  **/years** | **6/16** | **7/16** | **8/16** | **9/16** | **10/16** | **11/16** | **12/16** | **1/17** | **2/17** | **3/17** | **4/17** | **5/17** | **6/17** | **7/17** | **8/17** |
| --- | --- | --- | --- | --- | --- | --- | --- | --- | --- | --- | --- | --- | --- | --- | --- |
| 6/16 |  | 33.35 | 42.8 | 53.8 | 46.51 | 47.36 | 47.62 | 56.12 | 53.97 | 57.93 | 43.06 | 55 | 51.37 | 50.71 | 49.21 |
| 7/16 | **0.002** |  | 32.35 | 47.46 | 43.43 | 47.56 | 50.27 | 57.46 | 57.43 | 60.96 | 46.88 | 58.09 | 54.8 | 50.47 | 47.06 |
| 8/16 | **0.0022** | **0.0023** |  | 41.22 | 43.75 | 51.61 | 53.02 | 60.2 | 61.33 | 64.04 | 51.43 | 60.99 | 58.79 | 52.96 | 47.21 |
| 9/16 | **0.0026** | **0.0028** | **0.0013** |  | 41.33 | 47.22 | 52.45 | 55.33 | 55.36 | 55.3 | 50.17 | 54.85 | 53.32 | 49.02 | 48.02 |
| 10/16 | **0.002** | **0.0031** | **0.0013** | **0.0048** |  | 39.73 | 45.28 | 51.53 | 49.36 | 50.95 | 43.38 | 51.3 | 48.38 | 48.47 | 47.01 |
| 11/16 | **0.0028** | **0.0022** | **0.0025** | **0.0023** | **0.011** |  | 30.32 | 46.8 | 41.21 | 47.73 | 41.8 | 50.38 | 49.83 | 52.32 | 51.31 |
| 12/16 | **0.0024** | **0.002** | **0.0018** | **0.002** | **0.0018** | 0.0884 |  | 45.47 | 41.07 | 48.24 | 45.95 | 54.77 | 52.82 | 55.09 | 54.68 |
| 1/17 | **0.0021** | **0.0019** | **0.0031** | **0.0025** | **0.0024** | **0.0024** | **0.032** |  | 33.37 | 50.52 | 50.5 | 57.37 | 57.44 | 60.72 | 60.49 |
| 2/17 | **0.0018** | **0.0021** | **0.0021** | **0.0026** | **0.0028** | **0.002** | **0.011** | 0.087 |  | 34.33 | 47.31 | 54.69 | 53.79 | 58.25 | 59.35 |
| 3/17 | **0.0023** | **0.0017** | **0.0021** | **0.0028** | **0.0017** | **0.004** | **0.02** | **0.04** | **0.04** |  | 29.85 | 32.31 | 50.41 | 53.69 | 57.7 |
| 4/17 | **0.0025** | **0.0035** | **0.002** | **0.0023** | **0.0024** | **0.0027** | **0.0027** | **0.0024** | **0.0021** | 0.092 |  | 30.82 | 26.3 | 50.22 | 50.41 |
| 5/17 | **0.002** | **0.0015** | **0.0016** | **0.0024** | **0.0022** | **0.0029** | **0.0025** | **0.0027** | **0.0021** | 0.075 | 0.063 |  | 26.24 | 48.86 | 52.69 |
| 6/17 | **0.0024** | **0.0021** | **0.003** | **0.0026** | **0.0019** | **0.002** | **0.003** | **0.0028** | **0.0024** | **0.0399** | 0.062 | 0.058 |  | 39.16 | 46.87 |
| 7/17 | **0.0027** | **0.0024** | **0.0029** | **0.0018** | **0.0024** | **0.0027** | **0.002** | **0.0022** | **0.0024** | **0.0019** | **0.0014** | **0.0017** | **0.036** |  | 28.2 |
| 8/17 | **0.0024** | **0.0019** | **0.0029** | **0.0022** | **0.027** | **0.0017** | **0.0023** | **0.0027** | **0.0026** | **0.0035** | **0.0034** | **0.0103** | **0.039** | 0.262 |  |
