# Supplementary material for: Spatio-temporal dynamics of bacterial communities in the shoreline of Laurentian great Lake Erie and Lake St. Clair’s large freshwater ecosystems
Source: BMC Microbiol. 2021 Sep 21;21:253. doi: 10.1186/s12866-021-02306-y (PMC8454060; doi:10.1186/s12866-021-02306-y)
Supplement: Supplementary file 16 — Additional file 16: Supplementary Table 8. Pairwise comparison of alpha diversity and Bray–Curtis dissimilarity PCo1 & 2 indexes among 15 months of sampling across 6 different locations. [file 12866_2021_2306_MOESM16_ESM.docx]

**Supplementary Table 8.** Pairwise comparison of alpha diversity and Bray–Curtis dissimilarity PCo1 & 2 indexes among 15 months of sampling across 6 different locations

| **Months/years** | | **Chao1** | **Shannon** | **PCo1** | **PCo2** |
| --- | --- | --- | --- | --- | --- |
| 6/16 | 7/16 | 1.000 | 1.000 | 1.000 | 1.000 |
|  | 8/16 | 1.000 | 1.000 | 1.000 | .886 |
|  | 9/16 | .163 | **.000** | .**022** | .183 |
|  | 10/16 | .198 | **.000** | .248 | .992 |
|  | 11/16 | 1.000 | .275 | .174 | 1.000 |
|  | 12/16 | 1.000 | .986 | .859 | 1.000 |
|  | 1/17 | **.008** | .458 | .057 | .921 |
|  | 2/17 | .104 | **.006** | **.020** | .981 |
|  | 3/17 | **.000** | **.000** | **.001** | 1.000 |
|  | 4/17 | **.000** | **.000** | .075 | 1.000 |
|  | 5/17 | **.000** | **.000** | **.000** | 1.000 |
|  | 6/17 | **.011** | **.000** | .152 | 1.000 |
|  | 7/17 | .554 | **.024** | .431 | .995 |
|  | 8/17 | .288 | **.030** | .520 | .977 |
| 7/16 | 6/16 | 1.000 | 1.000 | 1.000 | 1.000 |
|  | 8/16 | 1.000 | 1.000 | 1.000 | .999 |
|  | 9/16 | .094 | **.000** | **.014** | .675 |
|  | 10/16 | .117 | **.000** | .180 | 1.000 |
|  | 11/16 | .998 | .052 | .122 | .998 |
|  | 12/16 | .998 | .755 | .777 | .995 |
|  | 1/17 | **.003** | .115 | **.037** | .439 |
|  | 2/17 | .057 | **.000** | **.012** | .637 |
|  | 3/17 | **.000** | **.000** | **.001** | .997 |
|  | 4/17 | **.000** | **.000** | .050 | 1.000 |
|  | 5/17 | **.000** | **.000** | **.000** | 1.000 |
|  | 6/17 | **.005** | **.000** | .105 | 1.000 |
|  | 7/17 | .399 | **.002** | .334 | 1.000 |
|  | 8/17 | .180 | **.003** | .415 | 1.000 |
| 8/16 | 6/16 | 1.000 | 1.000 | 1.000 | .886 |
|  | 7/16 | 1.000 | 1.000 | 1.000 | .999 |
|  | 9/16 | **.022** | **.000** | **.014** | .997 |
|  | 10/16 | **.029** | **.001** | .179 | 1.000 |
|  | 11/16 | .954 | .362 | .122 | .696 |
|  | 12/16 | .947 | .995 | .777 | .625 |
|  | 1/17 | **.000** | .562 | **.037** | **.047** |
|  | 2/17 | **.012** | **.011** | **.012** | .100 |
|  | 3/17 | **.000** | **.000** | **.001** | .682 |
|  | 4/17 | **.000** | **.000** | .050 | .932 |
|  | 5/17 | **.000** | **.000** | **.000** | .965 |
|  | 6/17 | **.001** | **.001** | .105 | .994 |
|  | 7/17 | .145 | **.038** | .334 | 1.000 |
|  | 8/17 | **.049** | **.047** | .415 | 1.000 |
| 9/16 | 6/16 | .163 | **.000** | **.022** | .183 |
|  | 7/16 | .094 | **.000** | **.014** | .675 |
|  | 8/16 | **.022** | **.000** | **.014** | .997 |
|  | 10/16 | 1.000 | 1.000 | 1.000 | .931 |
|  | 11/16 | .735 | .339 | 1.000 | .080 |
|  | 12/16 | .754 | **.007** | .835 | **.061** |
|  | 1/17 | 1.000 | .188 | 1.000 | **.001** |
|  | 2/17 | 1.000 | .989 | 1.000 | **.003** |
|  | 3/17 | .858 | 1.000 | 1.000 | .076 |
|  | 4/17 | .901 | 1.000 | 1.000 | .239 |
|  | 5/17 | **.019** | .982 | .904 | .312 |
|  | 6/17 | 1.000 | 1.000 | 1.000 | .492 |
|  | 7/17 | 1.000 | .915 | .994 | .912 |
|  | 8/17 | 1.000 | .888 | .986 | .970 |
| 10/16 | 6/16 | .198 | **.000** | .248 | .992 |
|  | 7/16 | .117 | **.000** | .180 | 1.000 |
|  | 8/16 | **.029** | **.001** | .179 | 1.000 |
|  | 9/16 | 1.000 | 1.000 | 1.000 | .931 |
|  | 11/16 | .788 | .779 | 1.000 | .942 |
|  | 12/16 | .805 | .058 | 1.000 | .911 |
|  | 1/17 | .999 | .583 | 1.000 | .171 |
|  | 2/17 | 1.000 | 1.000 | 1.000 | .304 |
|  | 3/17 | .815 | .982 | .899 | .937 |
|  | 4/17 | .866 | 1.000 | 1.000 | .997 |
|  | 5/17 | **.015** | .753 | .318 | .999 |
|  | 6/17 | 1.000 | 1.000 | 1.000 | 1.000 |
|  | 7/17 | 1.000 | .999 | 1.000 | 1.000 |
|  | 8/17 | 1.000 | .997 | 1.000 | 1.000 |
| 11/16 | 6/16 | 1.000 | .275 | .174 | 1.000 |
|  | 7/16 | .998 | .052 | .122 | .998 |
|  | 8/16 | .954 | .362 | .122 | .696 |
|  | 9/16 | .735 | .339 | 1.000 | .080 |
|  | 10/16 | .788 | .779 | 1.000 | .942 |
|  | 12/16 | 1.000 | .989 | .998 | 1.000 |
|  | 1/17 | .126 | 1.000 | 1.000 | .987 |
|  | 2/17 | .610 | .993 | 1.000 | .999 |
|  | 3/17 | **.009** | **.046** | .951 | 1.000 |
|  | 4/17 | **.012** | .498 | 1.000 | 1.000 |
|  | 5/17 | **.000** | **.006** | .423 | 1.000 |
|  | 6/17 | .166 | .802 | 1.000 | 1.000 |
|  | 7/17 | .982 | 1.000 | 1.000 | .956 |
|  | 8/17 | .879 | 1.000 | 1.000 | .884 |
| 12/16 | 6/16 | 1.000 | .986 | .859 | 1.000 |
|  | 7/16 | .998 | .755 | .777 | .995 |
|  | 8/16 | .947 | .995 | .777 | .625 |
|  | 9/16 | .754 | **.007** | .835 | .061 |
|  | 10/16 | .805 | .058 | 1.000 | .911 |
|  | 11/16 | 1.000 | .989 | .998 | 1.000 |
|  | 1/17 | .136 | .999 | .956 | .994 |
|  | 2/17 | .631 | .339 | .819 | 1.000 |
|  | 3/17 | **.010** | **.000** | .296 | 1.000 |
|  | 4/17 | **.013** | **.017** | .975 | 1.000 |
|  | 5/17 | **.000** | **.000** | **.030** | 1.000 |
|  | 6/17 | .178 | .066 | .997 | 1.000 |
|  | 7/17 | .985 | .609 | 1.000 | .930 |
|  | 8/17 | .892 | .661 | 1.000 | .837 |
| 1/17 | 6/16 | **.008** | .458 | .057 | .921 |
|  | 7/16 | **.003** | .115 | **.037** | .439 |
|  | 8/16 | **.000** | .562 | **.037** | **.047** |
|  | 9/16 | 1.000 | .188 | 1.000 | **.001** |
|  | 10/16 | .999 | .583 | 1.000 | .171 |
|  | 11/16 | .126 | 1.000 | 1.000 | .987 |
|  | 12/16 | .136 | .999 | .956 | .994 |
|  | 2/17 | 1.000 | .960 | 1.000 | 1.000 |
|  | 3/17 | 1.000 | **.019** | .997 | .989 |
|  | 4/17 | 1.000 | .307 | 1.000 | .872 |
|  | 5/17 | .295 | **.002** | .732 | .802 |
|  | 6/17 | 1.000 | .612 | 1.000 | .621 |
|  | 7/17 | .941 | .997 | 1.000 | .194 |
|  | 8/17 | .995 | .998 | .999 | .115 |
| 2/17 | 6/16 | .104 | **.006** | .020 | .981 |
|  | 7/16 | .057 | **.000** | .012 | .637 |
|  | 8/16 | **.012** | **.011** | .012 | .100 |
|  | 9/16 | 1.000 | .989 | 1.000 | .003 |
|  | 10/16 | 1.000 | 1.000 | 1.000 | .304 |
|  | 11/16 | .610 | .993 | 1.000 | .999 |
|  | 12/16 | .631 | .339 | .819 | 1.000 |
|  | 1/17 | 1.000 | .960 | 1.000 | 1.000 |
|  | 3/17 | .929 | .685 | 1.000 | .999 |
|  | 4/17 | .956 | .998 | 1.000 | .962 |
|  | 5/17 | **.034** | .258 | .915 | .927 |
|  | 6/17 | 1.000 | 1.000 | 1.000 | .803 |
|  | 7/17 | 1.000 | 1.000 | .993 | .338 |
|  | 8/17 | 1.000 | 1.000 | .983 | .217 |
| 3/17 | 6/16 | **.000** | **.000** | .001 | 1.000 |
|  | 7/16 | **.000** | **.000** | .001 | .997 |
|  | 8/16 | **.000** | **.000** | .001 | .682 |
|  | 9/16 | .858 | 1.000 | 1.000 | .076 |
|  | 10/16 | .815 | .982 | .899 | .937 |
|  | 11/16 | **.009** | **.046** | .951 | 1.000 |
|  | 12/16 | **.010** | **.000** | .296 | 1.000 |
|  | 1/17 | 1.000 | **.019** | .997 | .989 |
|  | 2/17 | .929 | .685 | 1.000 | .999 |
|  | 4/17 | 1.000 | .999 | .994 | 1.000 |
|  | 5/17 | .868 | 1.000 | 1.000 | 1.000 |
|  | 6/17 | 1.000 | .977 | .963 | 1.000 |
|  | 7/17 | .422 | .408 | .736 | .952 |
|  | 8/17 | .705 | .360 | .650 | .875 |
| 4/17 | 6/16 | **.000** | **.000** | .075 | 1.000 |
|  | 7/16 | **.000** | **.000** | .050 | 1.000 |
|  | 8/16 | **.000** | **.000** | .050 | .932 |
|  | 9/16 | .901 | 1.000 | 1.000 | .239 |
|  | 10/16 | .866 | 1.000 | 1.000 | .997 |
|  | 11/16 | **.012** | .498 | 1.000 | 1.000 |
|  | 12/16 | **.013** | **.017** | .975 | 1.000 |
|  | 1/17 | 1.000 | .307 | 1.000 | .872 |
|  | 2/17 | .956 | .998 | 1.000 | .962 |
|  | 3/17 | 1.000 | .999 | .994 | 1.000 |
|  | 5/17 | .818 | .939 | .662 | 1.000 |
|  | 6/17 | 1.000 | 1.000 | 1.000 | 1.000 |
|  | 7/17 | .492 | .972 | 1.000 | .998 |
|  | 8/17 | .770 | .959 | 1.000 | .990 |
| 5/17 | 6/16 | **.000** | **.000** | .000 | 1.000 |
|  | 7/16 | **.000** | **.000** | .000 | 1.000 |
|  | 8/16 | **.000** | **.000** | .000 | .965 |
|  | 9/16 | **.019** | .982 | .904 | .312 |
|  | 10/16 | **.015** | .753 | .318 | .999 |
|  | 11/16 | **.000** | **.006** | .423 | 1.000 |
|  | 12/16 | **.000** | **.000** | .030 | 1.000 |
|  | 1/17 | .295 | **.002** | .732 | .802 |
|  | 2/17 | **.034** | .258 | .915 | .927 |
|  | 3/17 | .868 | 1.000 | 1.000 | 1.000 |
|  | 4/17 | .818 | .939 | .662 | 1.000 |
|  | 6/17 | .234 | .727 | .463 | 1.000 |
|  | 7/17 | **.002** | .104 | .169 | 1.000 |
|  | 8/17 | **.008** | .086 | .125 | .996 |
| 6/17 | 6/16 | **.011** | **.000** | .152 | 1.000 |
|  | 7/16 | **.005** | **.000** | .105 | 1.000 |
|  | 8/16 | **.001** | **.001** | .105 | .994 |
|  | 9/16 | 1.000 | 1.000 | 1.000 | .492 |
|  | 10/16 | 1.000 | 1.000 | 1.000 | 1.000 |
|  | 11/16 | .166 | .802 | 1.000 | 1.000 |
|  | 12/16 | .178 | .066 | .997 | 1.000 |
|  | 1/17 | 1.000 | .612 | 1.000 | .621 |
|  | 2/17 | 1.000 | 1.000 | 1.000 | .803 |
|  | 3/17 | 1.000 | .977 | .963 | 1.000 |
|  | 4/17 | 1.000 | 1.000 | 1.000 | 1.000 |
|  | 5/17 | .234 | .727 | .463 | 1.000 |
|  | 7/17 | .966 | .999 | 1.000 | 1.000 |
|  | 8/17 | .998 | .998 | 1.000 | 1.000 |
| 7/17 | 6/16 | .554 | **.024** | .431 | .995 |
|  | 7/16 | .399 | **.002** | .334 | 1.000 |
|  | 8/16 | .145 | **.038** | .334 | 1.000 |
|  | 9/16 | 1.000 | .915 | .994 | .912 |
|  | 10/16 | 1.000 | .999 | 1.000 | 1.000 |
|  | 11/16 | .982 | 1.000 | 1.000 | .956 |
|  | 12/16 | .985 | .609 | 1.000 | .930 |
|  | 1/17 | .941 | .997 | 1.000 | .194 |
|  | 2/17 | 1.000 | 1.000 | .993 | .338 |
|  | 3/17 | .422 | .408 | .736 | .952 |
|  | 4/17 | .492 | .972 | 1.000 | .998 |
|  | 5/17 | **.002** | .104 | .169 | 1.000 |
|  | 6/17 | .966 | .999 | 1.000 | 1.000 |
|  | 8/17 | 1.000 | 1.000 | 1.000 | 1.000 |
| 8/17 | 6/16 | .288 | **.030** | .520 | .977 |
|  | 7/16 | .180 | **.003** | .415 | 1.000 |
|  | 8/16 | **.049** | **.047** | .415 | 1.000 |
|  | 9/16 | 1.000 | .888 | .986 | .970 |
|  | 10/16 | 1.000 | .997 | 1.000 | 1.000 |
|  | 11/16 | .879 | 1.000 | 1.000 | .884 |
|  | 12/16 | .892 | .661 | 1.000 | .837 |
|  | 1/17 | .995 | .998 | .999 | .115 |
|  | 2/17 | 1.000 | 1.000 | .983 | .217 |
|  | 3/17 | .705 | .360 | .650 | .875 |
|  | 4/17 | .770 | .959 | 1.000 | .990 |
|  | 5/17 | **.008** | .086 | .125 | .996 |
|  | 6/17 | .998 | .998 | 1.000 | 1.000 |
|  | 7/17 | 1.000 | 1.000 | 1.000 | 1.000 |

**Statistical analysis R code**

**GLMM full model; Chao 1 as variable**

Lch <-lmer (chao 1~ lake + (1 | rep) , (1 | beach/lake), data = data, REML = FALSE)

anova (Lch)

r.squaredGLMM (Lch)

Mch <-lmer (chao ~ month + (1 | rep) , (1 | week/month), data = data, REML = FALSE)

anova (Mch)

r.squaredGLMM (Mch)

**GLMM for each lake; Chao 1 as variable**

Lch <-lmer (chao ~ (month)/week + beach + (month)/week * beach + 1 | rep) , data = data, REML = FALSE)

anova (Lch)

r.squaredGLMM (Lch)

r.squaredGLMM (Mch)

**LMM on individual OTUs**

LMM1<- lmer(OTU1~Beach+Month + Beach*Month + (1|Weeks) , data=OTUtable)

anova (LMM1)

summary (LMM1)
